# A proteomic analysis of mushroom polysaccharide-treated HepG2 cells

**DOI:** 10.1038/srep23565

**Published:** 2016-03-29

**Authors:** Yangyang Chai, Guibin Wang, Lili Fan, Min Zhao

**Affiliations:** 1 College of Life Sciences, Northeast Forestry University, Harbin, PR China; 2 Northeast Institute of Geography and Agroecology, Chinese Academy of Sciences,Harbin, PR China

## Abstract

The anti-tumor properties of fungal polysaccharides have gained significant recognition in Asia and tropical America. In this study, the differential expression of proteins in normal HepG2 cells and those treated with polysaccharides that had been isolated from *Phellinus linteus* (PL), *Ganoderma lucidum* (GL) and *Auricularia auricula* (AA) was investigated. Using two-dimensional electrophoresis (2DE), a total of 104 protein spots were determined to be overexpressed in these cells compared with noncancerous regions. A total of 59 differentially expressed proteins were identified through MALDI-TOF-MS. In addition, 400 biological processes (BP), 133 cell components (CC) and 146 molecular functions (MF) were enriched by Gene Ontology (GO) analysis, and 78 KEGG pathways were enriched by pathway enrichment. Protein-Protein Interaction (PPI) analysis demonstrated the interaction networks affected by polysaccharides in HepG2 cells. Then, DJ-1 and 14-3-3 were identified as the key proteins in the networks, and the expression of the mRNA and proteins were evaluated using Real-time quantitative PCR (qRT-PCR) and Western blotting (WB), respectively. The results were in agreement with the 2DE. These results provided information on significant proteins of hepatocellular carcinoma (HCC) and form an important basis for the future development of valuable medicinal mushroom resources.

Hepatocellular carcinoma (HCC) is a common form of tumor worldwide. The evidence suggests that the incidence of HCC is rising, and it has become a major health problem. Its multistage process involves multiple factors in its etiology and many gene-environment interactions, including infection with hepatitis B or C (HBV or HCV), and ingestion of aflatoxin-contaminated food, and alcohol^1^. The development of HCC is associated with multiple changes at the messenger RNA (mRNA) and/or protein levels, some of which serve as tumor markers, e.g., glypican-3 (GPC3)^2^ (gi|23271174), α-fetoprotein^3^ (gi|178236), and less specifically, cyclin D1^4^ (gi|23273807) and the proliferating cell nuclear antigen^5^.

Medicinal mushrooms are among a number of well-known agents in Asian countries that have been taken orally since ancient times to combat viral and bacterial infections. It has been well-established that many commonly used compounds extracted from mushrooms act as immune modulators or as biological response modifiers (BRMs)^6^,^7^,^8^. We recently isolated the polysaccharides from *Phellinus linteus* (PL), *Ganoderma lucidum* (GL), and *Auriculari auricula* (AA) and investigated the molecular mechanisms underlying the anti-tumor properties of these polysaccharides in human liver cancer cells. We demonstrated that polysaccharides have antiproliferative effects in HepG2 and Bel-7404 human hepatoma cells. The growth inhibition of HepG2 and Bel-7404 cells by PL, GL, and AA is mediated through the induction of apoptosis and through G_1_- or S-phase cell cycle arrest. The mechanisms for the arrest involve the suppression of AKT (gi|63102175) activity via the inhibition of AKT phosphorylation at Thr^308^ and/or Ser^473^, the activation of Bcl-2 (gi|179371) family proteins, an increase in mitochondrial cytochrome C (gi|11128019) and Smac (gi|9454219) release, an enhancement in the expression of p27^Kip^ (gi|2982673) or p21^Cip^ (gi|453135), and the suppression of the activities of cyclin D1/CDK4 (gi|4502735) and cyclin E (gi|6630609)/CDK2 (gi|312803)^9^. However, the effects of mushroom polysaccharides on the identification of tumor markers in HepG2 cells have not been investigated. Proteomic studies of clinical tumor samples have led to the identification of cancer-specific protein markers, and these provide a basis for the development of new methods for the early diagnosis and early detection of cancers and may provide clues to improve our understanding of the molecular mechanism of cancer progression. Bioinformatics is an important technology that supports proteomics not only by providing an efficient means of analysis of the protein data but also by comprehensively evaluating functions of the known or new proteins. The technology includes Gene Ontology (GO) analysis, pathway enrichment and Protein-Protein Interaction (PPI) analysis. To identify the proteins and markers from HepG2 cells that were induced by PL, GL, and AA, a proteomic and bioinformatic approach was used. A number of proteins were separated by two-dimensional electrophoresis (2DE) and identified using mass spectrometry. All of the differentially expressed proteins were analyzed using bioinformatic technology. This analysis included the systematic cataloging of the protein expression levels at a large scale. Such studies may help to provide significant molecular targets in cancer progression and may have tremendous meaning for the utilization of valuable medicinal mushroom resources and the development of natural anti-tumor foods.

## Results

### Overview of the analysis of the protein expression profiles of the samples

Our previous experiments had proved that PL, GL, and AA had obvious inhibitory effects on HepG2 cells and induced their apoptosis^9^. We therefore subjected PL-, GL-, and AA-treated HepG2 cells to proteomics analyses. To ensure the quality and reproducibility of the results, the 2DE was performed at least three times under the same conditions.

**Figure 1:**
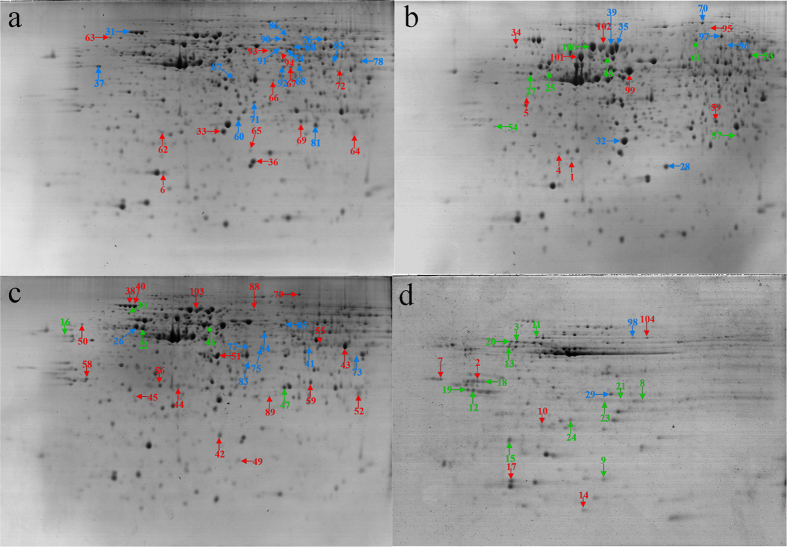
Comparison of a 2DE image of control HepG2 cells with those of the mushroom polysaccharide-treated HepG2 cells. (**a**) A 2DE image of control HepG2 cells. (**b**) A 2DE image of HepG2 cells treated with the PL polysaccharides. (**c**) A 2DE image of HepG2 cells treated with the GL polysaccharides. (**d**) A 2DE image of HepG2 cells treated with the AA polysaccharides. The red, blue and green symbols indicate differentially expressed proteins, down-regulated proteins, and up-regulated proteins, respectively.

2DE gel separation of the proteins from 12 pairs of HepG2 cells treated or not treated with PL, GL, and AA was performed. A series of 2DE maps for the soluble fraction proteins was constructed. Representative 2DE gel images following the silver staining of control HepG2 cells (Fig. 1a) or those treated with PL, GL, and AA (Fig. 1b–d) were produced using UMAX PowerLook 2100XL (Willich, Germany). The comparison of the differential protein expression between the control and treated cells shown in the 2DE images was performed using Image Master 2D Platinum software (Version 5.0, GE Healthcare). Under the same conditions, each gel contained approximately 656 ± 23 protein spots on average, and a total of 104 differentially expressed protein spots was detected.

The resulting identified spots were mapped onto the analytical gels, which were stained with Coomassie brilliant blue (CBB R-250). Based on these analyses, 104 differentially expressed protein spots were excised and subjected to in-gel digestion followed by peptide mass fingerprinting for protein identification. The criteria used to accept the identifications included the extent of sequence coverage, the number of peptides matched, the probability score, and whether the human protein appeared as one of the top candidates in the first-pass search, in which no restrictions were applied to the species of origin.

**Figure 2:**
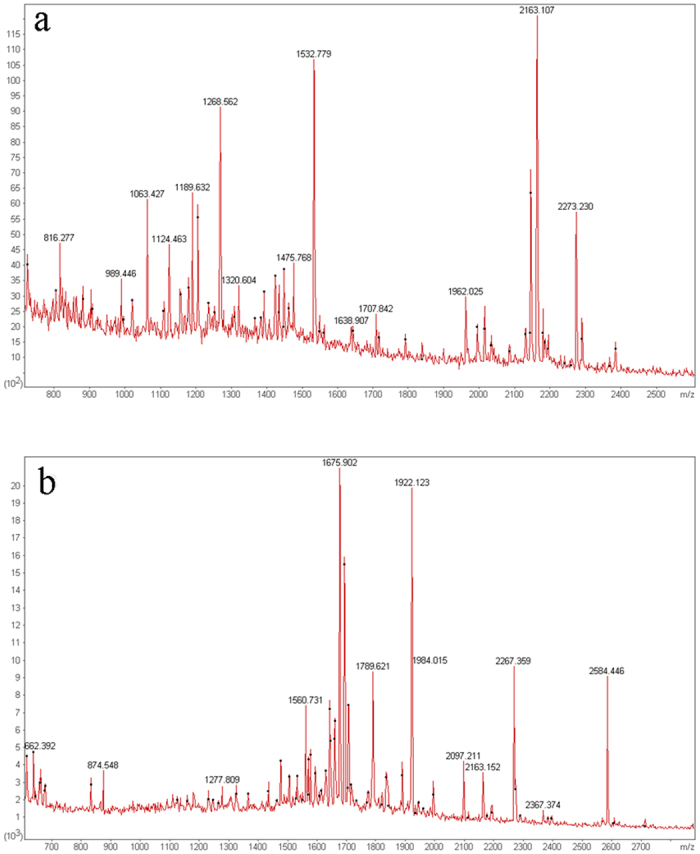
The representative MALDI-TOF-MS maps. (**a**) PMF of 14-3-3. (**b**) PMF of DJ-1.

The NCBInr human database (Human, 244004 sequences) was searched using the MASCOT software, and 59 differentially expressed proteins were evaluated by MALDI-TOF-MS mass spectrometry analysis. The results of the identification are summarized in Table 1. For each identified protein, a probability-based score greater than 75 was considered significant (P < 0.05). The identifications of spots No. 12 (Fig. 2a) and No. 96 (Fig. 2b) are shown as an example.

### GO, Pathway enrichment and PPI analysis

**Figure 3:**
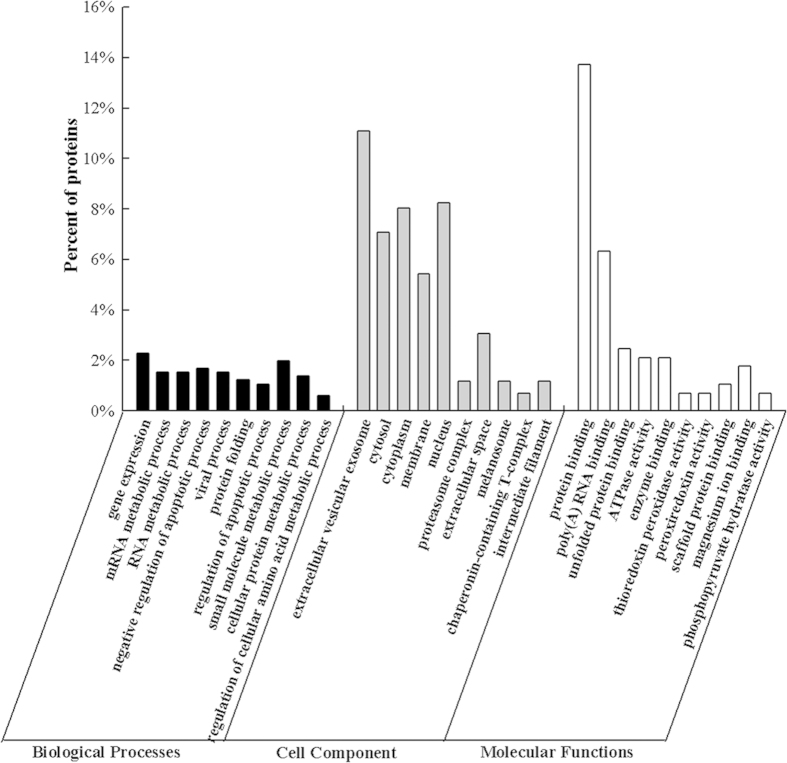
Gene Ontology classifications of proteins differentially expressed between normal HepG2 cells and the cells treated with the polysaccharides. The differentially expressed proteins were grouped into three hierarchically structured GO terms: biological process, cellular component, and molecular function.

The 59 differentially expressed proteins identified by the MASCOT analysis were subjected to Gene Ontology analysis (GO). This analysis identified 400 biological processes (BP), 133 cell components (CC) and 146 molecular functions (MF) that were enriched for this dataset(Supplementary Table S1). Of these 259 BP, 66 CC and 88 MF had P-values < 0.05. As seen in Fig. 3, the BP analysis showed that 2.28% of the identified proteins were involved in the gene expression process, while 1.98% of the proteins were associated with small molecule metabolic processes. Another 1.67% of the proteins were involved in the negative regulation of apoptotic processes. The CC analysis revealed that most of the identified proteins were distributed in the extracellular vesicular exosomes, cytosol, cytoplasm and membranes. The MF analysis demonstrated that 13.73% of the identified proteins had protein binding activity, and 6.34% of them had poly(A) RNA binding activity. The remaining proteins had various binding activities, including ribosome binding activity, cytokine binding activity, transcription factor binding activity and others. The up-regulated protein 14-3-3 (gi|5803227) and the down-regulated protein DJ-1 (gi|31543380) were mainly distributed in the extracellular vesicular exosomes, combining function and involved in apoptotic process.

In further analyses of the biological pathways, 78 KEGG pathways were enriched for this dataset (Supplementary Table S2). The top 10 pathways (P < 0.05) included antigen processing and presentation, proteasome, Epstein-Barr virus infection, protein processing in endoplasmic reticulum, glycolysis/gluconeogenesis, RNA degradation, amoebiasis, spliceosome, Legionellosis and pathogenic *Escherichia coli* infection (Table 2). A number of metabolic pathways were changed in HepG2 cells treated with polysaccharides. Three of the most enriched pathways were Epstein-Barr virus infection, protein processing in endoplasmic reticulum and antigen processing and presentation. There were 7, 6, and 5 proteins involved in these pathways, respectively.

**Figure 4:**
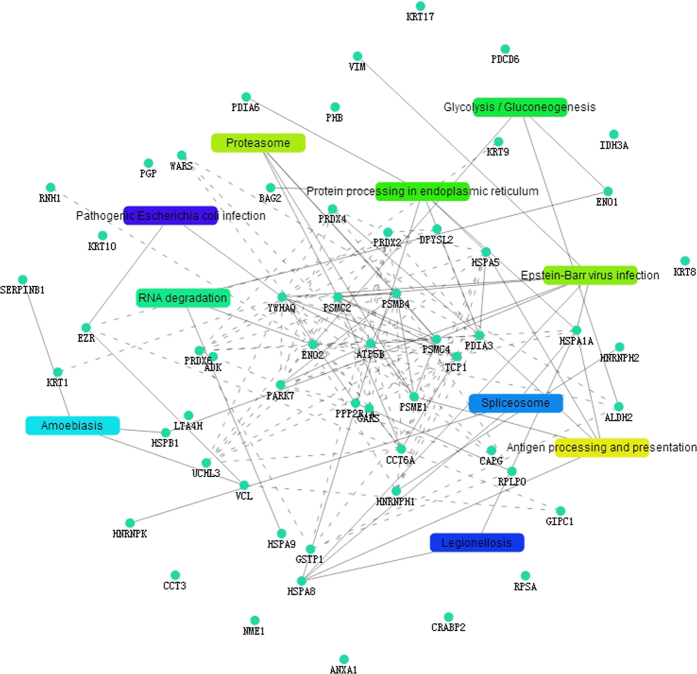
The protein-protein interaction networks of the differentially expressed proteins in STRING database. The signal pathways affected by the mushroom polysaccharides were clustered according to the network analysis. The solid lines represent direct interactions between proteins, and the dotted lines indicate indirect interactions between proteins.

The PPI analysis evaluated the interaction networks affected by the polysaccharides in HepG2 cells. These networks involved 59 proteins and 10 KEGG pathways (Fig. 4). The interactions of the proteins in these networks can be either direct or indirect. The nodes represent the proteins, and the lines between the nodes indicated direct or indirect protein-protein interaction modes. In the results of the PPI analysis, several proteins that directly interact with 14-3-3 were identified: t-complex polypeptide 1 (gi|36796), ATP synthase subunit beta (gi|179279) and phosphatase 2A regulatory subunit (gi|189428), whereas the protein DJ-1, the 60S acidic ribosomal protein P0 (gi|16933546), the prosome beta-subunit and others were identified as indirectly interacting proteins. Thus, up-regulation 14-3-3 may indirectly affect the down-regulation of protein DJ-1 in HepG2 cells treated with polysaccharide.

### Identification by Real-time quantitative PCR and Western blotting

**Figure 5:**
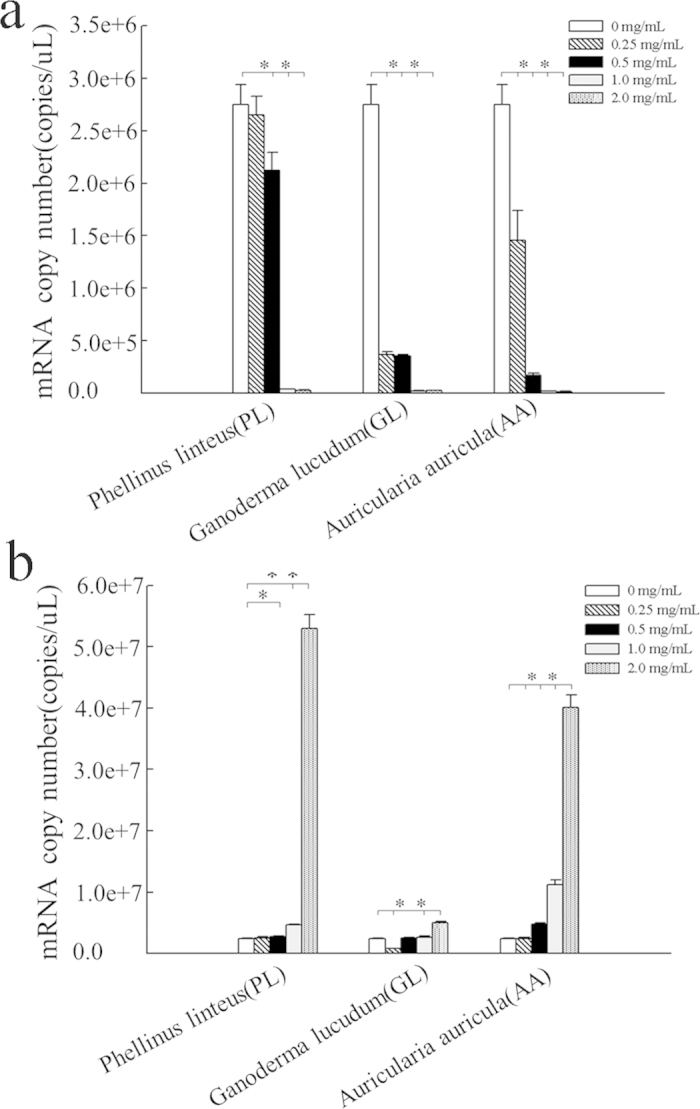
Quantitative Real-time PCR analysis of the expression of the DJ-1 and 14-3-3 mRNA in the normal HepG2 cells and the cells treated with the PL, GL and AA polysaccharides. (**a**) Gene expression of DJ-1. (**b**) Gene expression of 14-3-3. Gene expression is normalized to β-actin expression. The data represent the mean ± SD. *P < 0.05, **P < 0.01.

On the basis of the PPI results, two important proteins (DJ-1 and 14-3-3) were selected from 59 differentially expressed proteins for further study. Absolute quantification of the DJ-1 and 14-3-3 target genes was accomplished using Real-time quantitative PCR. The standard curve for DJ-1 showed an R^2^ = 0.999 and an amplification efficiency of 100.2%, and the standard curve for 14-3-3 displayed an R^2^ = 0.998 and an amplification efficiency of 104.9%. The two solubility curves, both of which comprised single peaks, showed no nonspecific amplification. The expression of the DJ-1 and 14-3-3 mRNA levels is shown in Fig. 5. Overall, the evaluation of the DJ-1 and 14-3-3 mRNA expressed in HepG2 cells showed that the mRNA expression of DJ-1 was down-regulated and that of 14-3-3 was up-regulated in the mushroom polysaccharide-treated HepG2 cells. Compared with the control HepG2 cells, a highly significant decrease in the DJ-1 expression level was observed at 0.25–2.0 mg/mL of the GL- and AA-treated groups, but in PL-treated group, highly significant decreases in the expression levels were observed at 0.5–2.0 mg/mL, but there was no significant difference at 0.25 mg/mL. However, the expression levels of 14-3-3 mRNA were generally elevated. The significance of the difference among the three groups varied. Compared with the control HepG2 cells, in the AA-treated group, the increases in the 14-3-3 expression level were highly significant at 0.5–2.0 mg/mL, whereas the increases in the expression levels were highly significant at 0.25 mg/mL and 1-2 mg/mL in the GL-treated group. In the PL-treated group, highly significant increases in the expression levels were observed at 1–2 mg/mL and a significant difference was found at 0.5 mg/mL, but the difference at 0.25 mg/mL was not significant. The changes in the mRNA levels were for both proteins were consistent with the proteomics results.

**Figure 6:**
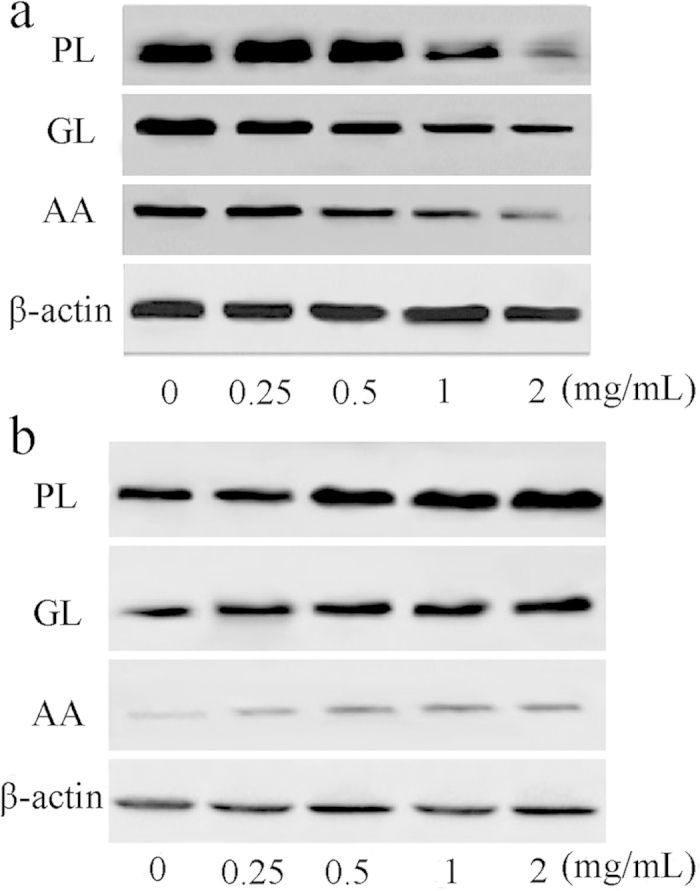
Expression of the DJ-1 and 14-3-3 proteins in normal HepG2 cells and the cells treated with the PL, GL and AA polysaccharides. (**a**) The protein expression of DJ-1 by Western blotting analysis. (**b**) The protein expression of 14-3-3 by Western blotting analysis. The protein expression was normalized to β-actin expression.

To evaluate the protein expression, HepG2 cells were treated with PL, GL, or AA, and β-actin was used as a control for the Western blotting analyses. The expression levels of DJ-1 in all of the groups were down-regulated, and the magnitude of the reduction increased with increasing concentration (Fig. 6a). However, 14-3-3 was up-regulated with increasing concentrations of the polysaccharides. HepG2 cells treated with GL and AA showed a trend toward increases, whereas HepG2 cells treated with PL showed little change in this protein (Fig. 6b). The statistical analysis showed that the results were consistent with the proteomics and qRT-PCR results.

### Discussion and Conclusion

PL, GL, and AA are basidiomycete fungi located mainly in Asia and tropical America. These fungi have gained significant recognition as medicinal mushrooms in traditional Oriental medicine. A large number of studies have shown that these fungal polysaccharides are associated with effects on immune function regulation^10^, anti-mutagenic activity^11^, and liver fibrosis inhibition^12^. Currently, many fungal polysaccharides are present in functional foods and drugs that are used for adjuvant cancer therapy, including lentinan^13^, *Coriolus versicolor* polysaccharides^14^, and *Polyporus* polysaccharides^15^. Fungal polysaccharides, particularly PL, are widely found in China, and their pharmacological effects have been widely proven^16^,^17^. Due to their broad potential applications in functional health foodstuffs, studies of fungal polysaccharides are of considerable value in the development of these foods.

This study was based on an ongoing proteomic and bioinformatic analysis of HepG2 cells treated with polysaccharides with the aim of screening protein markers for the diagnosis of HCC. The combination of two-dimensional electrophoresis and mass spectrometry is the most effective way to study complex patterns of protein expression^18^. Subsequent bioinformatic analyses can identify the target proteins or genes^19^. The measurement of the protein expression patterns of normal and diseased tissues or cell populations will lead to the characterization of diagnostic and prognostic markers. These data can be further employed for the analysis of the disease stages and which may also have an impact on the development of future therapies^20^. Thus, small clusters of proteins are preferred as representing ideal diagnostic markers that would enable an easier and more accurate diagnosis of the diseases and the potential for improved therapy^21^,^22^.

In the preparation of the 2DE maps presented in this study, tissue samples from separate individuals were used without pooling the samples. The total homogenates were fractionated by ultracentrifugation into their soluble fractions^23^. To minimize the influence of the methodology, we attempted, whenever possible, to ensure the similarity of the 2DE-PAGE protocols. Our initial analyses of twelve samples indicated that the overall protein pattern remained very similar across the samples. The 2DE pattern of the control and treated cells revealed a number of polypeptides that are associated with HCC, i.e., these polypeptides were expressed in HepG2 cells treated with polysaccharides but were absent in the normal HepG2 cells.

We identified 59 protein spots that were expressed in HepG2 cells treated with the polysaccharides but were absent in the normal HepG2 cells. Of the 59 identified proteins, spots 12 and 96 were present as multiple spots on the 2DE gels. The GO and KEGG pathway analyses are the most reliable methods to provide a better understanding of the BP, CC and MF of the target proteins^24^. The differentially expressed proteins were classified into different functional categories according to the GO analysis. These categories included gene expression, mRNA metabolic process, extracellular vesicular exosomes, cytosol, cytoplasm, protein binding, poly(A) RNA binding and others. The KEGG pathway analysis showed that antigen processing and presentation pathway (hsa04612), proteasome pathway (hsa03050) and Epstein-Barr virus infection (hsa05169) were the top three pathways with P < 0.05. We found that 14-3-3 was involved in many KEGG pathways, some of which were closely related with tumor markers and cellular signal transduction, including Epstein-Barr virus infection pathway, Hippo signaling pathway, viral carcinogenesis pathway, cell cycle pathway and PI3K-AKT signaling pathway. This result implies that resistance mechanisms associated with metabolism are important in HepG2 treated with polysaccharides.

In this study, we identified the protein interaction networks included in cellular functions and metabolisms, which are associated with cellular signal transduction closely, some important proteins, including ATP synthase subunit beta, vimentin and heat shock 70 kDa protein 1 A/1B also appeared in the biological networks. These proteins could interact with each other and together affect the expression of proteins in the polysaccharide-treated HepG2 cells. Additionally, these proteins were also closely related to the tumor biomarkers^25^,^26^,^27^ for HCC. Therefore, the PPI results revealed that several signal pathways were affected by the polysaccharides, and some of these may be identified and serve as diagnostic and prognostic markers in HCC^28^,^29^.

DJ-1 was first identified as a novel candidate oncogene product that transforms mouse NIH3T3 cells in cooperation with activated ras^30^. The genomic DNA of both human and mouse DJ-1 comprises seven exons, and exons 2–7 encode the DJ-1 proteins. The human DJ-1 gene maps to chromosome 1p36.2-p36.3, which represents a hot spot of chromosome abnormalities that have been found in several tumors^31^. DJ-1 is preferentially expressed in the testes and moderately expressed in other tissues, and it is translocated from the cytoplasm to the nucleus during the cell cycle after mitogen stimulation. These observations suggest that DJ-1 has a growth-related function. Liu et al. found that the DJ-1 protein is clearly up-regulated in HCC tissues^32^. However, in the present study, the DJ-1 protein was down-regulated in the PL-treated HepG2 cells. A recent study found that DJ-1 was down-regulated in the *Radix Rehmanniae Preparata* polysaccharide-treated in hippocampus of rats^33^. The DJ-1 protein participates in a number of apoptotic pathways, and the apoptosis is inhibited by preventing the oxidative damage by free radicals^34^. DJ-1 can prevent TRAIL-induced apoptosis by inhibiting the formation of the death-inducing signaling complex (DISC)^35^. To prevent apoptosis, DJ-1 and the Fas death domain-associated protein (Daxx) (gi|48146287) combine in the nucleus, thus preventing Daxx from associating with ASK1 (gi|5174547) and undergoing a translocation from the nucleus to the cytoplasm^36^. The DJ-1 protein negatively regulate the activity of PTEN (gi|4240387)^37^. Therefore, a lower expression of DJ-1 can decrease the phosphorylation of PKB/Akt, whereas a higher expression of DJ-1 can increase the PKB/Akt phosphorylation and cell survival^38^. Studies have shown that abnormal expression of DJ-1 plays an important role in the invasion and metastasis of HCC^39^,^40^. Under low oxygen conditions, the stability of the transcription factor HIF1 (gi|16611719) mainly depends on the PI3K/Akt/mTOR signaling pathways. The DJ-1-regulated expression of Akt and mTOR (gi|4826730) is crucial to maintaining the stability of HIF1. DJ-l can also regulate the activity of AMPK (gi|786491). The carcinogenic activity of DJ-1 regulates mTOR and AMPK functions as activated factor of HIF1 upstream function of cancer cells^41^. These findings suggest that the DJ-1 protein will be useful as a prognostic biomarker for HCC.

The 14-3-3 proteins are expressed in all eukaryotic cells, and their amino acid sequences are highly conserved from yeast to mammals. Seven isoforms encoded by seven distinct genes have been identified in mammals, more than 10 isoforms have been identified in plants, and two isoforms have been identified in yeast, Drosophila, and *C. elegans*. Interestingly, the yeast 14-3-3 genes are functionally interchangeable with the plant and mammalian isoforms, which indicates a high level of functional conservation of the gene products. The 14-3-3 proteins assemble as stable homo- and heterodimers^42^,^43^,^44^,^45^. All of the 14-3-3 proteins appear to share similar tertiary structures, which were first defined for the τ and ζ isoforms^46^. The 14-3-3 proteins are a family of conserved regulatory molecules that are expressed in all eukaryotic cells. A striking feature of the 14-3-3 proteins is their ability to bind a multitude of functionally diverse signaling proteins, including kinases, phosphatases, and transmembrane receptors. This plethora of interacting proteins allows 14-3-3 to play important roles in a wide range of vital regulatory processes, including mitogenic signal transduction, apoptotic cell death, and cell cycle control. Recent studies have suggested that 14-3-3 may inhibit apoptosis and is involved in tumor genesis and development, and its protein or gene is usually abnormally expressed in a variety of human malignancies. Additional studies on the 14-3-3σ (gi|5454052) protein show that this protein can exhibit an upward or downward trend during the development of tumors. In addition, the 14-3-3σ protein has become a new molecular marker and a new drug target for the treatment of malignant tumors, which forms the basis for important theoretical and practical knowledge. In this review, we examined the interactions between 14-3-3 and other proteins, including ATP synthase subunit beta, DJ-1, keratin and others, and signaling pathways that involve 14-3-3, and discussed the expression of 14-3-3 in polysaccharide-treated HepG2 cells.

In conclusion, the mechanisms for the polysaccharide-treated effects in are complex. Our data showed the changes in the expression of only a few of the differentially expressed proteins and the results of the polysaccharide-treated effects on PPI. Further basic and clinical investigations will be needed to determine whether these proteins can be used as markers for HCC and to develop natural anti-tumor foods.

## Methods

### Cells and reagents

The PL, GL, and AA powders were purchased from Zhoushan-Tech (Shanghai, China) and purified using ethanol precipitation methods followed by DEAE-cellulose and gel permeation chromatography^47^. The purified component was single fraction. The human HCC cell line HepG2 (American Type Culture Collection ATCC) was cultured in RPMI 1640 medium supplemented with 10% heat-inactivated fetal calf serum (Hyclone, Thermo Scientific, USA), 100 units mL^−1^ penicillin, 100 g mL^−1^ streptomycin at 37 °C in an atmosphere containing 5% CO_2_. Carbamide, sulfocarbamide, DTT, CHAPS, NL IPG buffer (pH 4–7), and IPG gels were purchased from Amresco. Anti-mouse antibodies (Cell Signaling), including anti-DJ-1 and anti-14-3-3 protein primary antibodies and secondary antibody, β-actin, a Real-time quantitative PCR kit (Platinum^®^ SYBR^®^ Green qPCR SuperMix-UDG; Invitrogen), TRIzol reagent (Invitrogen) and TIANScript II RT Kit (TIANGEN, Beijing, China) were used for the verification tests.

### 2DE and gel analysis

HepG2 cells were treated with PL, GL, or AA (1 mg ml^−1^) for 72 h, and the whole-cell extracts were lysed in RIPA buffer (Thermo Scientific, USA). The samples were prepared for 2DE^48^. The first-dimension isoelectric focusing was conducted using a Multiphor II system as described by the manufacturer (Amersham Bioscience Inc.). A precast immobilized pH gradient (IPG) strip (24 cm, pH 4–7, linear gradient) was used for the first-dimensional separation. Samples containing between 700 mg to 1,000 mg total protein were loaded onto an IPG strip, allowed to swell for 16 h and then rehydrated for 24 h. The isoelectric focusing was conducted at 250 V for 3 h, at 500 V for 2 h, followed by 1 h at 1,000 V, a gradient to 10,000 V for 3 h, and then from 10,000 V up to 130,000 V for the pH 4-7 strips. All of the IEF steps were conducted at 20 °C. After the first-dimensional IEF, the IPG gel strips were placed in an equilibration solution (6 mol L^−1^ urea, 200 g L^−1^ SDS, 300 g L^−1^ glycerol, and 50 mol L^−1^ Tris-HCl, pH 8.8) containing 10 g L^−1^ dithiothreitol and shaken for 15 min. The gels were then transferred to an equilibration solution containing 25 g L^−1^ iodoacetamide to alkylate the thiols, shaken for 15 min, and then placed on a 125 g L^−1^ polyacrylamide gel slab. The separation in the second dimension was conducted using a Tris-glycine buffer containing 1 g L^−1^ SDS at a current setting of 5 mA gel^−1^ for the initial 0.5 h and at 18 mA gel^−1^ thereafter; the temperature was maintained at 20 °C.The experiments were carried out in triplicate.

The SDS-PAGE gels were visualized by the modified CBB R-250 staining method. The analytical gels were scanned at a resolution of 300 dpi (dots per inch), and the image analysis was performed with ImageMaster 2D Platinum Software (Version 5.0, GE Healthcare) following the user’s manual. The apparent molecular weight of each protein in the gel was determined using protein markers.

### In-gel protein digestion

The proteins were digested in-gel with bovine trypsin (modified sequencing grade, Roche Molecular Biochemicals) as previously described^49^. The gel spots of interest were manually excised, washed twice with 200 μL of distilled water at room temperature for 30 min, washed twice with 200 μL of destaining solution (50% acetonitrile (ACN) and 50 mM ammonium acetate, pH 7.0) at room temperature for 30 min, washed twice with 100 μL of 100% acetonitrile for 5 min, and air-dried. The gel pieces were then rehydrated with 10 μL of trypsin digestion solution (10 ng μL^−1^ trypsin in 25 mM ammonium bicarbonate, pH 8.5) at 4 °C for 1 h and covered with 8 μL of digestion buffer (1 mM calcium chloride and 25 mM ammonium bicarbonate, pH 8.5) at 37 °C for 16 h. After the digestion, the protein peptides were collected, and the minced gels were extracted three times with extraction bufferI (0.1% trifluoroacetic acid, TFA), extraction bufferII (30%ACN, 0.1% TFA) and extraction bufferIII (60% ACN, 0.1% TFA). After each extraction, the samples were centrifuged at 1,000 *x*g for 20 s, and all of the supernatants were combined, vacuum dried, and stored at −80 °C until analysis by mass spectrometry (MS).

The samples weremixed with the matrix solution of α-cyano-4-hydroxycinnamic acid (Bruker-Daltonics, Billerica, MA, USA) in 50% acetonitrile and 0.1% TFA for 30 min. Then, 1.5 μL of the reconstituted in-gel digest sample followed by 1 μL of the matrix solution were spotted on the Anchor chip target plate (600/384 F, Bruker-Daltonics). The dried sample on the target plate was washed twice with 1 μL of 0.1% TFA, incubated for 30 s, and dried for MALDI-TOF-MS analysis.

### MALDI-TOF-MS analysis

For protein identification, the dried spots were analyzed using a REFLEX-III (Bruker) MALDI-TOF-MS^50^. The spectrometer was run in the positive ion mode and in the reflector mode with the following settings: pulsed N2 laser 337 nm, ion source 1 = 19.00 kV, ion source 2 = 16.50 kV, reflector voltage = 20.00 kV, lens voltage = 8.80 kV, pulsed ion extraction time = 80 ns, matrix suppression = 400 Da, and positive reflector mode. At least three different areas of each protein sample on the MALDI target were selected for analysis, and each area was analyzed five times; the averages were used as the standard peak parameters. The MS images were analyzed using X-TOF/X-MASS software (Version 3.2, Bruker-Daltonics, Billerica, MA, USA). The measured tryptic peptide masses were transferred through the MS BioTools program (Bruker-Daltonics) as inputs to search against the taxonomy of Homo in the nonredundant NCBI database (http://www.ncbi.nlm.nih.gov/) using MASCOT software (Version 2.2, Matrix Science, London, UK). Good matches were classified as those having a Mascot score higher than 75 (threshold).

### Protein data bioinformatic analysis

The differentially expressed proteins were subjected to GO analysis, pathway enrichment and PPI analysis. The GO analysis was performed against the DAVID database (http://david.abcc.ncifcrf.gov/) and included biological process enrichment (BP), cell component enrichment (CC) and molecular function enrichment (MF). The pathway enrichment used the KEGG database (http://www.kegg.jp/) and analyzed the significance of pathway. Base on the interactions among the pathways in the KEGG database, the PPI was built using the STRING database (http://string.embl.de/).

### Real-time quantitative PCR assay

The total RNA was extracted using TRIzol reagent and reverse transcribed into the first-strand cDNA using a TIANScript II RT Kit. A control containing no reverse transcriptase was included to confirm the absence of contaminating DNA. The successfully reverse transcribed cDNA was diluted ten-fold and used as the template for the Real-time quantitative PCR (qRT-PCR). The primer sequences are shown below:

DJ-1 (forward), 5′-CGCGGATCCCATGGCTTCCAAAAGAGCT-3′;

DJ-1 (reverse), 5′-CCCGAATTCCTAGTCTTTAAGAACAAG-3′;

14-3-3 (forward), 5′-AAATGTTGTAGGAGCCCGTAGG-3′;

14-3-3 (reverse), 5′-GAAGCATTGGGGATCAAGAACT-3′.

The qRT-PCR was performed using the following temperature conditions: 30 s at 95 °C and 35 cycles of 5 s at 95 °C, 15 s at 58 °C and 20 s at 68 °C, and one final cycle of 15 s at 95 °C, 1 min at 60 °C and 15 s at 95 °C. β-actin was used as the internal control gene, and SYBR Green I was used as fluorochrome. Finally, the coefficient of variation was calculated. The samples had the same mRNA concentration.

### Western blot assay

HepG2 cells were cultured until the cells reached the logarithmic phase of growth and were then treated with PL, GL, or AA. The medium was discarded after the cells had been cultured for 72 h. The cells were washed twice with PBS at 4 °C. The cells in each 25-cm^2^ culture flask were lysed in 0.5 mL of RIPA buffer containing 10 μL of protease inhibitors (Thermo) for 5 min on ice. The lysates were then collected and centrifuged at 2640 *x*g for 5 min. The BCA protein kit was used to determine the total protein content.

The samples were lysed in 5 × buffer and boiled for 5 min, then cooled and applied to a PAGE gel. The voltage for the first gel was 80 V, and the voltage for the second gel was 120 V. The gel electrophoresis was terminated when samples had migrated to approximately 1 cm from the bottom of the PAGE gel. The size of the NC membrane was the same as that of the PAGE gel. To ensure a minimum number of air bubbles, every step was performed submerged in the transfer buffer. The PAGE gel, NC membrane, the filter plate, and a spongy cushion were layered in that order and subjected to electrophoresis at 15 V for 30 min. The NC membrane was washed three times with TBST for 10 min, and 5% skim milk was then added. The membrane was then incubated overnight at 4 °C. The NC membrane was washed three times with TBST for 10 min. The membrane was then incubated with the primary antibody, which was diluted 1:100 in TBS, for 1 h and then washed three times with TBST for 10 min. The membrane was then incubated with the secondary antibody diluted in TBS for 1 h, washed three times with TBST for 10 min, and developed with a WB ultra-sensitive light-emitting liquid using an LAS-3000 imaging system (FUJIFILM, Japan).

### Statistical analysis of the data

The experiments were carried out in triplicate. The data are expressed as the means ± standard error of mean (SEM) and were statistically tested by performing *t*-tests and analysis of variance (ANOVA) using SPSS 19.0 software (Chicago, IL, USA). P < 0.05 was considered statistically significant.

## Additional Information

**How to cite this article**: Chai, Y. *et al.* A Proteomic Analysis of Mushroom Polysaccharide-Treated HepG2 Cells. *Sci. Rep.* **6**, 23565; doi: 10.1038/srep23565 (2016).

## Supplementary Material

Supplementary Information

## Figures and Tables

**Table 1:** MS identification of differentially expressed protein spots in mushroom polysaccharide-treated HepG2 cells.

Spots number	Protein name	gi number	matched peptiders	MALDI-TOF-MS sequence coverage	Protein scoreb	MW (Dalton)	PI
3	ATP synthase subunit beta	32189394	22	47%	125	56525	5.26
8	heat shock protein beta-1	4504517	10	43%	94	22826	5.98
9	cellular retinoic acid-binding protein 2	4503029	12	68%	103	15854	5.42
11	vimentin	62414289	38	73%	217	53676	5.06
12	14-3-3	5803227	18	59%	109	28032	4.68
13	neurone-specific enolase	930063	25	62%	210	47467	4.94
15	hCG1985580, isoform CRA_c	119571372	9	63%	109	14498	5.05
16	RNH1 protein	15029922	12	38%	86	50104	4.83
18	Keratin 10	21961605	13	28%	95	59020	5.09
19	keratin, type I cytoskeletal 9	55956899	17	41%	94	62255	5.14
20	vimentin variant 3	167887751	24	52%	140	49680	5.19
21	heat shock protein 27	662841	14	67%	151	22427	7.83
22	protein disulfide isomerase	1710248	21	65%	239	46512	4.95
23	Peroxiredoxin-4	49456297	11	45%	113	30742	5.86
24	thiol-specific antioxidant proteins	1617118	12	72%	192	18486	5.19
25	Tat binding protein 7	263099	21	56%	165	51633	5.52
26	mitochondrial ATP synthase	89574029	28	61%	215	48083	4.95
27	keratin, type I cytoskeletal 17	4557701	42	66%	361	48361	4.97
28	nucleoside diphosphate kinase A	38045913	18	80%	160	19869	5.42
29	prohibitin	46360168	24	88%	224	29859	5.57
30	phosphatase 2A regulatory subunit	189428	21	33%	145	65232	5.1
31	Glucose-regulated protein precursor	386758	26	40%	272	72185	5.03
32	glutathione S-transferase P	332837089	14	63%	127	23555	5.43
35	Stress-70 protein, mitochondrial	24234688	40	55%	279	73920	5.89
37	40S ribosomal protein SA	9845502	19	53%	143	32947	4.79
39	Heat shock 70 kDa protein 1A/1B	167466173	31	63%	245	70294	5.48
41	PDZ domain-containing protein	5031715	16	47%	159	36141	5.9
46	keratin 8	119617057	44	62%	236	57829	5.41
47	peroxiredoxin-6	4758638	11	44%	104	25133	6
48	heterogeneous nuclear ribonucleoprotein K	119583084	23	55%	171	49002	5.46
53	chaperonin containing TCP1, subunit 6A isoform a variant	62089036	31	64%	171	58239	6.25
54	ubiquitin carboxyl-terminal hydrolase isozyme L3BAG family molecular	5174741	13	53%	121	26337	4.84
57	chaperone regulator 2	4757834	13	51%	110	23928	6.25
60	prosome beta-subunit	551547	14	58%	102	25950	5.7
61	glycyltRNAsynthetase	1311463	22	35%	122	83828	6.61
68	leukocyte elastase inhibitor	13489087	10	35%	100	42829	5.9
70	Vinculin	24657579	26	32%	185	117234	5.83
71	proteasome activator complex subunit 1	5453990	21	65%	199	28876	5.78
73	annexin A1	119582950	13	41%	98	40475	6.57
74	Heterogeneous nuclear ribonucleoprotein H	48145673	21	52%	118	49384	5.79
75	Isocitrate dehydrogenase	5031777	13	28%	97	40022	6.47
76	Chaperonin containing TCP1, subunit 3	14124984	30	46%	233	60934	6.1
77	keratin 1	11935049	18	34%	88	66198	8.16
78	adenosine kinase isoform a	32484973	26	67%	189	39078	6.23
80	Chain A, Structure Of Human Tryptophanyl-TrnaSynthetase in Complex With Trna	112489952	13	36%	103	44408	7.26
81	dihydropyrimidinase-related protein 2 isoform 2	4503377	29	68%	233	62711	5.95
82	Chain A, Crystal Structure Of Human Enolase 1	203282367	16	41%	96	47350	6.99
83	phosphoglycolate phosphatase	108796653	12	38%	99	34441	5.85
84	26S protease regulatory subunit 7 isoform 1	4506209	31	64%	231	49002	5.71
85	heterogeneous nuclear ribonucleoprotein H2	9624998	26	44%	170	49517	5.89
86	leukotriene A-4 hydrolase	4505029	23	40%	158	69868	5.8
87	60S acidic ribosomal protein P0	4506667	20	57%	137	34423	5.71
90	t-complex polypeptide 1	36796	19	39%	129	60869	6.03
91	aldehyde dehydrogenase, mitochondrial	25777732	17	37%	90	56859	6.33
92	macrophage-capping protein isoform 2	371502127	14	41%	78	37119	6.72
96	protein DJ-1	31543380	15	70%	129	20050	6.33
97	Ezrin	46249758	43	58%	290	69313	5.94
98	protein disulfide isomerase family A, member 3	119597640	27	51%	172	54454	6.78
100	heat shock cognate 71 kDa protein isoform 1	5729877	28	48%	209	71082	5.37

**Table 2:** Significantly enriched KEGG pathways of differentially expressed proteins.

	Pathway name	ID	Genes	Count	P-value
1	Antigen processing and presentation	hsa04612	P08107,P30101,P11021,Q06323,P11142	5	7.53e-05
2	Proteasome	hsa03050	P35998,P28070,Q06323,P43686	4	8.88e-05
3	Epstein-Barr virus infection	hsa05169	P08107,P04792,P35998,P08670,P27348,P11142,P43686	7	9.31e-05
4	Protein processing in endoplasmic reticulum	hsa04141	P08107,P30101,O95816,Q15084,P11021,P11142	6	2.67e-04
5	Glycolysis/Gluconeogenesis	hsa00010	P09104,P06733,P05091	3	5.48e-03
6	RNA degradation	hsa03018	P38646,P09104,P06733	3	7.25e-03
7	Amoebiasis	hsa05146	P30740,P04792,P18206	3	2.14e-02
8	Spliceosome	hsa03040	P08107,P61978,P11142	3	3.44e-02
9	Legionellosis	hsa05134	P08107,P11142	2	3.62e-02
10	Pathogenic Escherichia coli infection	hsa05130	P15311,P27348	2	3.62e-02
