# Supplementary material for: A proteomic analysis of mushroom polysaccharide-treated HepG2 cells
Source: Sci Rep. 2016 Mar 29;6:23565. doi: 10.1038/srep23565 (PMC4810362; doi:10.1038/srep23565)
Supplement: Supplementary Information [file srep23565-s1.pdf]

SUPPLEMENTARY INFORMATION

A Proteomic Analysis of Mushroom Polysaccharide-Treated HepG2 Cells

Yangyang Chai[1,*], Guibin Wang[1,*], Lili Fan[2], Min Zhao[1]

[1] College of Life Sciences, Northeast Forestry University, Harbin, PR China

[2] Northeast Institute of Geography and Agroecology, Chinese Academy of Sciences, Harbin, PR China

[*] These authors contributed equally to this work. Correspondence and requests for materials should be addressed to M.Z. (email: 82191513@163.com; Tel. /fax: +86 0451 82191513)

Supplementary Table S1: The GO enrichment analysis of differentially expressed proteins
Supplementary Table S2: The KEGG pathway analysis of differentially expressed proteins

Table S1 The GO enrichment analysis of differentially expressed proteins

Table S1A Biological Brocesses (BP) of GO enrichment analysis

GO Name	GO ID	Genes	Count	P-value
gene expression	GO:0010467	P08107,P23381,P04792,P35998,P55795,P61978,P31943,P05388,P30153,P28070,P41250,P08865,Q06323,P11142,P43686	15	4.41e-14
mRNA metabolic process	GO:0016071	P08107,P04792,P35998,P05388,P30153,P28070,P08865,Q06323,P11142,P43686	10	1.29e-12
RNA metabolic process	GO:0016070	P08107,P04792,P35998,P05388,P30153,P28070,P08865,Q06323,P11142,P43686	10	3.70e-12
negative regulation of apoptotic process	GO:0043066	P08107,P04792,P35998,P32119,P38646,P04083,P11021,P28070,P09211,Q06323,P43686	11	3.66e-09
viral process	GO:0016032	P35998,P08670,P61978,P05388,P28070,P08865,Q06323,P05787,P11142,P43686	10	2.58e-08
protein folding	GO:0006457	P30101,P40227,P49368,P17987,O95816,Q15084,P38646,P11142	8	3.35e-08
regulation of apoptotic process	GO:0042981	P15531,P35232,P35998,P32119,P28070,Q06323,P43686	7	3.89e-08
small molecule metabolic process	GO:0044281	P55263,P15531,P35998,P09960,P50213,P09104,P28070,P09211,P06576,P06733,P05091,Q06323,P43686	13	1.20e-07
cellular protein metabolic process	GO:0044267	P30101,P40227,P49368,P17987,Q15084,P05388,P38646,P11021,P08865	9	1.32e-07
regulation of cellular amino acid metabolic process	GO:0006521	P35998,P28070,Q06323,P43686	4	1.05e-06
intermediate filament organization	GO:0045109	P35527,P08670,Q04695	3	1.13e-06
antigen processing and presentation of exogenous peptide antigen via MHC class I, TAP-dependent	GO:0002479	P30101,P35998,P28070,Q06323,P43686	5	2.27e-06
antigen processing and presentation of exogenous peptide antigen via MHC class I	GO:0042590	P30101,P35998,P28070,Q06323,P43686	5	2.69e-06
negative regulation of ubiquitin-protein ligase activity involved in mitotic cell cycle	GO:0051436	P35998,P28070,Q06323,P43686	4	2.80e-06
DNA damage response, signal transduction by p53 class mediator resulting in cell cycle arrest	GO:0006977	P35998,P28070,Q06323,P43686	4	3.77e-06
positive regulation of ubiquitin-protein ligase activity involved in mitotic cell cycle	GO:0051437	P35998,P28070,Q06323,P43686	4	3.99e-06
regulation of ubiquitin-protein ligase activity involved in mitotic cell cycle	GO:0051439	P35998,P28070,Q06323,P43686	4	4.96e-06

anaphase-promoting complex-dependent proteasomal ubiquitin-dependent protein catabolic process	GO:0031145	P35998,P28070,Q06323,P43686	4	7.44e-06
antigen processing and presentation of peptide antigen via MHC class I	GO:0002474	P30101,P35998,P28070,Q06323,P43686	5	7.67e-06
regulation of angiogenesis	GO:0045765	P23381,P13489,P04264	3	1.24e-05
cellular component movement	GO:0006928	P04792,P08670,P04083,P18206	4	2.11e-05
'de novo' posttranslational protein folding	GO:0051084	P40227,P49368,P17987	3	2.76e-05
RNA splicing	GO:0008380	P55795,P61978,P31943,P30153,P11142	5	5.13e-05
response to unfolded protein	GO:0006986	P08107,P04792,P11142	3	5.84e-05
protein polyubiquitination	GO:0000209	P35998,P28070,Q06323,P43686	4	5.99e-05
binding of sperm to zona pellucida	GO:0007339	P40227,P49368,P17987	3	6.57e-05
apoptotic process	GO:0006915	P35998,P08670,P27348,P30153,P28070,Q06323,P43686	7	7.65e-05
negative regulation of cell growth	GO:0030308	P08107,P35232,P30153,P06733	4	1.00e-04
negative regulation of acute inflammatory response	GO:0002674	P04083,P09211	2	1.43e-04
ATP catabolic process	GO:0006200	P08107,P35998,P11021,P06576,P11142,P43686	6	1.47e-04
G1/S transition of mitotic cell cycle	GO:0000082	P35998,P28070,Q06323,P43686	4	1.57e-04
cellular nitrogen compound metabolic process	GO:0034641	P35998,P28070,Q06323,P43686	4	1.71e-04
protein refolding	GO:0042026	P08107,P11142	2	2.26e-04
mRNA catabolic process	GO:0006402	P08107,P13489	2	2.92e-04
epidermis development	GO:0008544	P35527,P29373,Q04695	3	3.14e-04
morphogenesis of an epithelium	GO:0002009	P18206,Q04695	2	3.29e-04
negative regulation of protein kinase activity	GO:0006469	P04792,P09211,Q99497	3	3.70e-04
hydrogen peroxide catabolic process	GO:0042744	P32119,P30041	2	4.07e-04
mitotic cell cycle	GO:0000278	P35998,P30153,P28070,Q06323,P43686	5	4.48e-04
nuclear-transcribed mRNA catabolic process, nonsense-mediated decay	GO:0000184	P05388,P30153,P08865	3	7.78e-04
osteoblast differentiation	GO:0001649	P35232,P35998,P06576	3	1.00e-03
carbohydrate metabolic process	GO:0005975	P50213,P09104,A6NDG6,P06733,P05091	5	1.30e-03
negative regulation of male germ cell proliferation	GO:2000255	Q13162	1	1.49e-03
positive regulation of androgen receptor activity	GO:2000825	Q99497	1	1.49e-03
negative regulation of protein K48-linked deubiquitination	GO:1903094	Q99497	1	1.49e-03
negative regulation of TRAIL-activated apoptotic signaling pathway	GO:1903122	Q99497	1	1.49e-03

maintenance of protein localization in endoplasmic reticulum	GO:0035437	P11021	1	1.49e-03
negative regulation of leukocyte proliferation	GO:0070664	P09211	1	1.49e-03
negative regulation of death-inducing signaling complex assembly	GO:1903073	Q99497	1	1.49e-03
glycyl-tRNA aminoacylation	GO:0006426	P41250	1	1.49e-03
positive regulation of oxidative phosphorylation uncoupler activity	GO:2000277	Q99497	1	1.49e-03
regulation of TRAIL receptor biosynthetic process	GO:0045560	Q99497	1	1.49e-03
negative regulation of fibril organization	GO:1902904	P11142	1	1.49e-03
neutrophil clearance	GO:0097350	P04083	1	1.49e-03
positive regulation of superoxide dismutase activity	GO:1901671	Q99497	1	1.49e-03
nitric oxide storage	GO:0035732	P09211	1	1.49e-03
negative regulation of ubiquitin-specific protease activity	GO:2000157	Q99497	1	1.49e-03
regulation of protein folding in endoplasmic reticulum	GO:0060904	P11021	1	1.49e-03
dATP biosynthetic process	GO:0006175	P55263	1	1.49e-03
response to virus	GO:0009615	P08107,P04792,P06733	3	1.54e-03
retina homeostasis	GO:0001895	P04792,P04264	2	1.64e-03
cellular response to oxidative stress	GO:0034599	P32119,Q99497	2	1.98e-03
cellular response to heat	GO:0034605	P08107,O75340	2	1.98e-03
substantia nigra development	GO:0021762	P27348,P11021	2	2.16e-03
response to oxidative stress	GO:0006979	P32119,P04264,P30041	3	2.29e-03
negative regulation of extrinsic apoptotic signaling pathway	GO:2001237	P09211,Q99497	2	2.76e-03
AMP salvage	GO:0044209	P55263	1	2.98e-03
negative regulation of protein acetylation	GO:1901984	Q99497	1	2.98e-03
regulation of lipid transport by positive regulation of transcription from RNA polymerase II promoter	GO:0072369	P61978	1	2.98e-03
dopamine uptake involved in synaptic transmission	GO:0051583	Q99497	1	2.98e-03
cerebellum structural organization	GO:0021589	P11021	1	2.98e-03
peptidyl-serine dephosphorylation	GO:0070262	P30153	1	2.98e-03
mRNA splicing, via spliceosome	GO:0000398	P55795,P61978,P31943	3	3.16e-03
negative regulation of cell death	GO:0060548	P08107,Q99497	2	3.30e-03
response to endoplasmic reticulum	GO:0034976	P30101,Q15084	2	3.30e-03

stress				
protein targeting	GO:0006605	P27348,O14908	2	3.53e-03
cellular response to hydrogen peroxide	GO:0070301	P04083,Q99497	2	4.14e-03
activation of signaling protein activity involved in unfolded protein response	GO:0006987	Q15084,P11021	2	4.27e-03
negative regulation of monocyte chemotactic protein-1 production	GO:0071638	P09211	1	4.46e-03
positive regulation of neutrophil apoptotic process	GO:0033031	P04083	1	4.46e-03
regulation of low-density lipoprotein particle clearance	GO:0010988	P61978	1	4.46e-03
gliogenesis	GO:0042063	P04083	1	4.46e-03
rRNA export from nucleus	GO:0006407	P08865	1	4.46e-03
negative regulation of cysteine-type endopeptidase activity involved in apoptotic signaling pathway	GO:2001268	Q99497	1	4.46e-03
cellular heat acclimation	GO:0070370	P08107	1	4.46e-03
endonucleolytic cleavage to generate mature 3'-end of SSU-rRNA from (SSU-rRNA, 5.8S rRNA, LSU-rRNA)	GO:0000461	P08865	1	4.46e-03
negative regulation of interleukin-8 secretion	GO:2000483	P04083	1	4.46e-03
common myeloid progenitor cell proliferation	GO:0035726	P09211	1	4.46e-03
keratinocyte differentiation	GO:0030216	P13645,P04083	2	4.53e-03
tRNA aminoacylation for protein translation	GO:0006418	P23381,P41250	2	4.80e-03
gluconeogenesis	GO:0006094	P09104,P06733	2	4.80e-03
signal transduction	GO:0007165	P30101,P35232,P29373,P61978,P04083,Q04695,Q16555	7	5.75e-03
positive regulation of low-density lipoprotein particle receptor biosynthetic process	GO:0045716	P61978	1	5.95e-03
protein metabolic process	GO:0019538	O95816	1	5.95e-03
diadenosine tetraphosphate biosynthetic process	GO:0015966	P41250	1	5.95e-03
negative regulation of nitric-oxide synthase biosynthetic process	GO:0051771	P09211	1	5.95e-03
positive regulation of endothelial cell chemotaxis by VEGF-activated vascular endothelial growth factor	GO:0038033	P04792	1	5.95e-03

receptor signaling pathway				
negative regulation of hydrogen peroxide-mediated programmed cell death	GO:1901299	Q99497	1	5.95e-03
nucleobase-containing small molecule metabolic process	GO:0055086	P55263,P15531	2	6.09e-03
viral transcription	GO:0019083	P05388,P08865	2	6.71e-03
platelet degranulation	GO:0002576	P11021,P18206	2	6.71e-03
negative regulation of tyrosine phosphorylation of Stat3 protein	GO:0042518	P30153	1	7.43e-03
negative regulation of biosynthetic process	GO:0009890	P09211	1	7.43e-03
vascular endothelial growth factor receptor-2 signaling pathway	GO:0036324	O75340	1	7.43e-03
positive regulation of endothelial cell chemotaxis	GO:2001028	P04792	1	7.43e-03
negative regulation of stress-activated MAPK cascade	GO:0032873	P09211	1	7.43e-03
membrane to membrane docking	GO:0022614	P15311	1	7.43e-03
hydrogen peroxide metabolic process	GO:0042743	Q99497	1	7.43e-03
endonucleolytic cleavage in ITS1 to separate SSU-rRNA from 5.8S rRNA and LSU-rRNA from tricistronic rRNA transcript (SSU-rRNA, 5.8S rRNA, LSU-rRNA)	GO:0000447	P08865	1	7.43e-03
negative regulation of myeloid leukocyte differentiation	GO:0002762	P15531	1	7.43e-03
Bergmann glial cell differentiation	GO:0060020	P08670	1	7.43e-03
peptide catabolic process	GO:0043171	P09960	1	7.43e-03
second-messenger-mediated signaling	GO:0019932	P30153	1	7.43e-03
negative regulation of cell proliferation	GO:0008285	P08107,P23381,P15531,P35232	4	8.05e-03
endoplasmic reticulum unfolded protein response	GO:0030968	Q15084,P11021	2	8.55e-03
progesterone receptor signaling pathway	GO:0050847	P35232	1	8.91e-03
alcohol metabolic process	GO:0006066	P05091	1	8.91e-03
response to hydrostatic pressure	GO:0051599	P05787	1	8.91e-03
negative regulation of glucocorticoid receptor signaling pathway	GO:2000323	P35232	1	8.91e-03

establishment or maintenance of apical/basal cell polarity	GO:0035088	P15311	1	8.91e-03
adenosine metabolic process	GO:0046085	P55263	1	8.91e-03
angiogenesis	GO:0001525	P23381,O75340,P06576	3	9.50e-03
translational termination	GO:0006415	P05388,P08865	2	9.83e-03
positive regulation of endopeptidase activity	GO:0010950	Q06323	1	1.04e-02
positive regulation of prostaglandin biosynthetic process	GO:0031394	P04083	1	1.04e-02
negative regulation of protein export from nucleus	GO:0046826	Q99497	1	1.04e-02
adherens junction assembly	GO:0034333	P18206	1	1.04e-02
negative regulation of inflammatory response to antigenic stimulus	GO:0002862	P28070	1	1.04e-02
positive regulation of hair follicle development	GO:0051798	Q04695	1	1.04e-02
ribosomal small subunit assembly	GO:0000028	P08865	1	1.04e-02
negative regulation of transcription, DNA-templated	GO:0045892	P35232,P27348,P06733,P11142	4	1.13e-02
pyrimidine nucleobase catabolic process	GO:0006208	Q16555	1	1.19e-02
negative regulation of inclusion body assembly	GO:0090084	P08107	1	1.19e-02
purine ribonucleoside salvage	GO:0006166	P55263	1	1.19e-02
complement activation, lectin pathway	GO:0001867	P04264	1	1.19e-02
regulation of stress-activated MAPK cascade	GO:0032872	P09211	1	1.19e-02
lens fiber cell development	GO:0070307	P08670	1	1.19e-02
negative regulation of gene expression	GO:0010629	P15531,Q99497	2	1.33e-02
cell differentiation involved in embryonic placenta development	GO:0060706	P05787	1	1.33e-02
regulation of intracellular pH	GO:0051453	P06576	1	1.33e-02
cellular response to interleukin-6	GO:0071354	P35232	1	1.33e-02
positive regulation of superoxide anion generation	GO:0032930	P09211	1	1.33e-02
ethanol catabolic process	GO:0006068	P05091	1	1.33e-02
regulation of androgen receptor signaling pathway	GO:0060765	Q99497	1	1.33e-02
viral life cycle	GO:0019058	P05388,P08865	2	1.35e-02
SRP-dependent cotranslational protein targeting to membrane	GO:0006614	P05388,P08865	2	1.35e-02
estrous cycle phase	GO:0060206	P04083	1	1.48e-02

mitotic nuclear envelope reassembly	GO:0007084	P30153	1	1.48e-02
type B pancreatic cell proliferation	GO:0044342	P55263	1	1.48e-02
ER overload response	GO:0006983	P11021	1	1.48e-02
negative regulation of tumor necrosis factor-mediated signaling pathway	GO:0010804	P09211	1	1.48e-02
negative regulation of transcription by competitive promoter binding	GO:0010944	P35232	1	1.48e-02
negative regulation of cell adhesion involved in substrate-bound cell migration	GO:0006933	P06576	1	1.48e-02
translational elongation	GO:0006414	P05388,P08865	2	1.60e-02
positive regulation of angiogenesis	GO:0045766	P04792,O75340	2	1.60e-02
RNA processing	GO:0006396	P61978,P31943	2	1.62e-02
positive regulation of protein homodimerization activity	GO:0090073	Q99497	1	1.63e-02
ribonucleoside monophosphate biosynthetic process	GO:0009156	P55263	1	1.63e-02
response to reactive oxygen species	GO:0000302	P09211	1	1.63e-02
positive regulation of glutamate secretion	GO:0014049	Q16555	1	1.63e-02
glycolytic process	GO:0006096	P09104,P06733	2	1.67e-02
negative regulation of catalytic activity	GO:0043086	P04083,P13489	2	1.76e-02
synaptic vesicle transport	GO:0048489	Q16555	1	1.77e-02
protein retention in ER lumen	GO:0006621	P30101	1	1.77e-02
negative regulation of oxidative stress-induced intrinsic apoptotic signaling pathway	GO:1902176	P04792	1	1.77e-02
protein complex assembly	GO:0006461	P40121,P30153	2	1.79e-02
inflammatory response	GO:0006954	P09960,P04083,Q99497	3	1.84e-02
negative regulation of vascular endothelial growth factor receptor signaling pathway	GO:0030948	O75340	1	1.92e-02
regulation of DNA replication	GO:0006275	P30153	1	1.92e-02
neutrophil homeostasis	GO:0001780	P04083	1	1.92e-02
DNA catabolic process	GO:0006308	P15531	1	1.92e-02
purine-containing compound salvage	GO:0043101	P55263	1	1.92e-02
positive regulation of receptor-mediated endocytosis	GO:0048260	P61978	1	1.92e-02
response to other organism	GO:0051707	P05787	1	1.92e-02
astrocyte development	GO:0014002	P08670	1	1.92e-02
xenobiotic metabolic process	GO:0006805	P09211,P05091	2	2.01e-02

membrane organization	GO:0061024	P27348,P11142	2	2.04e-02
cell redox homeostasis	GO:0045454	P30101,Q15084	2	2.04e-02
negative regulation of JUN kinase activity	GO:0043508	P09211	1	2.07e-02
negative regulation of androgen receptor signaling pathway	GO:0060766	P35232	1	2.07e-02
cytoskeletal anchoring at plasma membrane	GO:0007016	P15311	1	2.07e-02
regulation of Wnt signaling pathway	GO:0030111	P30153	1	2.07e-02
alpha-beta T cell differentiation	GO:0046632	P04083	1	2.07e-02
DNA biosynthetic process	GO:0071897	P35232	1	2.07e-02
glucose metabolic process	GO:0006006	P09104,P06733	2	2.14e-02
regulation of cell death	GO:0010941	P08107	1	2.21e-02
barbed-end actin filament capping	GO:0051016	P40121	1	2.21e-02
positive regulation of interleukin-1 beta production	GO:0032731	P04792	1	2.21e-02
establishment of endothelial barrier	GO:0061028	P15311	1	2.21e-02
tubulin complex assembly	GO:0007021	P17987	1	2.21e-02
cell projection assembly	GO:0030031	P40121	1	2.21e-02
arachidonic acid secretion	GO:0050482	P04083	1	2.21e-02
regulation of ERK1 and ERK2 cascade	GO:0070372	P09211	1	2.21e-02
cellular response to antibiotic	GO:0071236	P11021	1	2.36e-02
positive regulation of protein localization to nucleus	GO:1900182	Q99497	1	2.36e-02
positive regulation of tumor necrosis factor biosynthetic process	GO:0042535	P04792	1	2.36e-02
negative regulation of interleukin-1 beta production	GO:0032691	P09211	1	2.36e-02
mitochondrial ATP synthesis coupled proton transport	GO:0042776	P06576	1	2.36e-02
positive regulation of G1/S transition of mitotic cell cycle	GO:1900087	P04083	1	2.36e-02
neurotransmitter biosynthetic process	GO:0042136	P05091	1	2.36e-02
cellular metabolic process	GO:0044237	P50213,P06576	2	2.36e-02
axon guidance	GO:0007411	P15311,P11142,Q16555	3	2.36e-02
positive regulation of blood vessel endothelial cell migration	GO:0043536	P04792	1	2.50e-02
positive regulation of cardiac muscle hypertrophy	GO:0010613	P55263	1	2.50e-02
leukotriene metabolic process	GO:0006691	P09960	1	2.50e-02
regulation of I-kappaB kinase/NF-kappaB signaling	GO:0043122	P04792	1	2.50e-02

I-kappaB phosphorylation	GO:0007252	Q13162	1	2.50e-02
tryptophanyl-tRNA aminoacylation	GO:0006436	P23381	1	2.50e-02
endocytosis	GO:0006897	P15531,Q16555	2	2.64e-02
response to amine	GO:0014075	P15531	1	2.65e-02
negative regulation of proteasomal ubiquitin-dependent protein catabolic process	GO:0032435	O14908	1	2.65e-02
nucleobase-containing small molecule interconversion	GO:0015949	P15531	1	2.65e-02
hepatocyte apoptotic process	GO:0097284	P05787	1	2.79e-02
regulation of neuron differentiation	GO:0045664	Q16555	1	2.79e-02
synaptic transmission	GO:0007268	O14908,P05091,P11142	3	2.89e-02
retinoic acid metabolic process	GO:0042573	P29373	1	2.94e-02
hepatocyte differentiation	GO:0070365	P04083	1	2.94e-02
phospholipid catabolic process	GO:0009395	P30041	1	2.94e-02
protein localization to cell surface	GO:0034394	P18206	1	2.94e-02
negative regulation of MAPK cascade	GO:0043409	P09211	1	2.94e-02
cellular response to glucocorticoid stimulus	GO:0071385	P04083	1	2.94e-02
negative regulation of neuron death	GO:1901215	Q99497	1	2.94e-02
positive regulation of interleukin-8 production	GO:0032757	Q99497	1	3.08e-02
cellular response to fatty acid	GO:0071398	P15531	1	3.08e-02
ethanol oxidation	GO:0006069	P05091	1	3.08e-02
glutamate secretion	GO:0014047	O14908	1	3.23e-02
cerebellar Purkinje cell layer development	GO:0021680	P11021	1	3.23e-02
olfactory bulb development	GO:0021772	Q16555	1	3.37e-02
membrane depolarization	GO:0051899	Q99497	1	3.37e-02
regulation of cell differentiation	GO:0045595	P30153	1	3.52e-02
removal of superoxide radicals	GO:0019430	P32119	1	3.52e-02
cellular response to glucose starvation	GO:0042149	P11021	1	3.52e-02
ATP biosynthetic process	GO:0006754	P06576	1	3.52e-02
cellular response to vascular endothelial growth factor stimulus	GO:0035924	P04792	1	3.66e-02
positive regulation of vesicle fusion	GO:0031340	P04083	1	3.66e-02
regulation of neuron apoptotic process	GO:0043523	Q99497	1	3.80e-02
ceramide metabolic process	GO:0006672	P30153	1	3.80e-02
positive regulation of erythrocyte differentiation	GO:0045648	P08107	1	3.80e-02
negative regulation of neuron	GO:0043524	P32119,Q99497	2	3.83e-02

apoptotic process				
glutathione derivative biosynthetic process	GO:1901687	P09211	1	3.95e-02
apoptotic cell clearance	GO:0043277	Q15084	1	3.95e-02
spinal cord development	GO:0021510	Q16555	1	3.95e-02
positive regulation of protein insertion into mitochondrial membrane involved in apoptotic signaling pathway	GO:1900740	P27348	1	3.95e-02
membrane hyperpolarization	GO:0060081	Q99497	1	3.95e-02
positive regulation of transforming growth factor beta receptor signaling pathway	GO:0030511	O14908	1	3.95e-02
regulation of mitochondrial membrane potential	GO:0051881	Q99497	1	4.09e-02
platelet activation	GO:0030168	P11021,P18206	2	4.10e-02
translational initiation	GO:0006413	P05388,P08865	2	4.20e-02
leukotriene biosynthetic process	GO:0019370	P09960	1	4.23e-02
positive regulation of DNA binding	GO:0043388	P15531	1	4.23e-02
blastocyst development	GO:0001824	P43686	1	4.38e-02
reactive oxygen species metabolic process	GO:0072593	Q13162	1	4.52e-02
negative regulation of protein serine/threonine kinase activity	GO:0071901	P04792	1	4.52e-02
cell death	GO:0008219	P04792,P41250	2	4.73e-02
chaperone mediated protein folding requiring cofactor	GO:0051085	P11142	1	4.80e-02
response to X-ray	GO:0010165	P04083	1	4.80e-02
negative regulation of TOR signaling	GO:0032007	O75340	1	4.80e-02
epithelial cell-cell adhesion	GO:0090136	P18206	1	4.80e-02
apical junction assembly	GO:0043297	P18206	1	4.80e-02
translation	GO:0006412	P23381,P05388,P08865	3	5.08e-02
cellular response to interleukin-4	GO:0071353	P11021	1	5.09e-02
fibrinolysis	GO:0042730	P04264	1	5.09e-02
GTP biosynthetic process	GO:0006183	P15531	1	5.23e-02
purine nucleobase metabolic process	GO:0006144	P55263	1	5.23e-02
CTP biosynthetic process	GO:0006241	P15531	1	5.37e-02
UTP biosynthetic process	GO:0006228	P15531	1	5.37e-02
negative regulation of fibroblast proliferation	GO:0048147	P09211	1	5.37e-02
skin development	GO:0043588	P35527	1	5.37e-02
negative regulation of protein kinase B signaling	GO:0051898	O75340	1	5.51e-02

regulation of protein stability	GO:0031647	O14908	1	5.51e-02
inactivation of MAPK activity	GO:0000188	P30153	1	5.51e-02
muscle filament sliding	GO:0030049	P08670	1	5.51e-02
peptide cross-linking	GO:0018149	P04083	1	5.65e-02
proteolysis	GO:0006508	P30101,O75340,P09960,Q99497,P43686	5	5.76e-02
leukocyte cell-cell adhesion	GO:0007159	P15311	1	5.79e-02
receptor internalization	GO:0031623	P15311	1	5.79e-02
negative regulation of protein binding	GO:0032091	Q99497	1	5.79e-02
negative regulation of I-kappaB kinase/NF-kappaB signaling	GO:0043124	P09211	1	5.79e-02
extrinsic apoptotic signaling pathway	GO:0097191	P05787	1	5.93e-02
regulation of proteolysis	GO:0030162	P30740	1	5.93e-02
endothelial cell migration	GO:0043542	O14908	1	5.93e-02
positive regulation of endothelial cell migration	GO:0010595	O75340	1	5.93e-02
positive regulation of extrinsic apoptotic signaling pathway in absence of ligand	GO:2001241	P30153	1	5.93e-02
insulin secretion	GO:0030073	P04083	1	5.93e-02
response to interleukin-1	GO:0070555	P04083	1	6.07e-02
ER-associated ubiquitin-dependent protein catabolic process	GO:0030433	P11021	1	6.07e-02
positive regulation of extrinsic apoptotic signaling pathway	GO:2001238	P30101	1	6.07e-02
endocrine pancreas development	GO:0031018	P04083	1	6.07e-02
mitochondrion organization	GO:0007005	Q99497	1	6.07e-02
regulation of RNA splicing	GO:0043484	P31943	1	6.07e-02
response to testosterone	GO:0033574	P15531	1	6.21e-02
positive regulation of cytokinesis	GO:0032467	O14908	1	6.21e-02
negative regulation of tumor necrosis factor production	GO:0032720	P09211	1	6.35e-02
nucleoside diphosphate phosphorylation	GO:0006165	P15531	1	6.35e-02
protein export from nucleus	GO:0006611	P38646	1	6.49e-02
response to cocaine	GO:0042220	Q16555	1	6.49e-02
tumor necrosis factor-mediated signaling pathway	GO:0033209	P05787	1	6.49e-02
protein catabolic process	GO:0030163	P15374	1	6.77e-02
post-Golgi vesicle-mediated transport	GO:0006892	P11142	1	6.77e-02
cellular component disassembly involved in execution phase of	GO:0006921	P08670	1	6.91e-02

apoptosis				
cellular response to calcium ion	GO:0071277	P13645	1	6.91e-02
tricarboxylic acid cycle	GO:0006099	P50213	1	7.05e-02
filopodium assembly	GO:0046847	P15311	1	7.05e-02
response to amphetamine	GO:0001975	Q16555	1	7.19e-02
keratinization	GO:0031424	Q04695	1	7.19e-02
positive regulation of protein ubiquitination	GO:0031398	P11021	1	7.33e-02
response to organic substance	GO:0010033	P30153	1	7.33e-02
negative regulation of protein phosphorylation	GO:0001933	Q99497	1	7.33e-02
negative regulation of neuron projection development	GO:0010977	P08670	1	7.47e-02
single fertilization	GO:0007338	Q99497	1	7.47e-02
regulation of growth	GO:0040008	P30153	1	7.47e-02
generation of precursor metabolites and energy	GO:0006091	P06576	1	7.47e-02
sarcomere organization	GO:0045214	P05787	1	7.60e-02
ribosome biogenesis	GO:0042254	P05388	1	7.60e-02
regulation of translational initiation	GO:0006446	P04792	1	7.74e-02
regulation of cell adhesion	GO:0030155	P30153	1	7.74e-02
circadian regulation of gene expression	GO:0032922	P55263	1	7.74e-02
negative regulation of MAP kinase activity	GO:0043407	P09211	1	7.88e-02
embryonic forelimb morphogenesis	GO:0035115	P29373	1	8.02e-02
lamellipodium assembly	GO:0030032	P18206	1	8.02e-02
positive regulation of T cell proliferation	GO:0042102	P55263	1	8.15e-02
histone deacetylation	GO:0016575	P35232	1	8.43e-02
cellular response to drug	GO:0035690	P15531	1	8.56e-02
positive regulation of translation	GO:0045727	Q04695	1	8.70e-02
intrinsic apoptotic signaling pathway	GO:0097193	P27348	1	8.70e-02
negative regulation of ERK1 and ERK2 cascade	GO:0070373	P09211	1	8.70e-02
protein import into nucleus	GO:0006606	P30101	1	8.84e-02
protein targeting to mitochondrion	GO:0006626	P38646	1	8.84e-02
neurotransmitter secretion	GO:0007269	P11142	1	8.97e-02
actin filament bundle assembly	GO:0051017	P15311	1	8.97e-02
proton transport	GO:0015992	P06576	1	8.97e-02
ubiquitin-dependent protein catabolic process	GO:0006511	P35998,P15374	2	9.19e-02
arachidonic acid metabolic process	GO:0019369	P09960	1	9.52e-02

hippocampus development	GO:0021766	P15531	1	9.52e-02
glutathione metabolic process	GO:0006749	P09211	1	9.78e-02
cellular response to glucose stimulus	GO:0071333	P15531	1	1.01e-01
regulation of inflammatory response	GO:0050727	Q99497	1	1.01e-01
nucleobase-containing compound metabolic process	GO:0006139	Q16555	1	1.01e-01
positive regulation of endothelial cell proliferation	GO:0001938	O75340	1	1.02e-01
regulation of synaptic plasticity	GO:0048167	O14908	1	1.03e-01
lactation	GO:0007595	P15531	1	1.06e-01
negative regulation of extrinsic apoptotic signaling pathway in absence of ligand	GO:2001240	P08107	1	1.06e-01
response to peptide hormone	GO:0043434	P04083	1	1.10e-01
response to calcium ion	GO:0051592	O75340	1	1.11e-01
positive regulation of epithelial cell proliferation	GO:0050679	P15531	1	1.15e-01
negative regulation of transforming growth factor beta receptor signaling pathway	GO:0030512	P11021	1	1.18e-01
adult locomotory behavior	GO:0008344	Q99497	1	1.22e-01
positive regulation of cell growth	GO:0030307	Q04695	1	1.23e-01
chromosome segregation	GO:0007059	P30153	1	1.23e-01
ATP hydrolysis coupled proton transport	GO:0015991	P06576	1	1.24e-01
response to cAMP	GO:0051591	P15531	1	1.28e-01
activation of cysteine-type endopeptidase activity involved in apoptotic process	GO:0006919	O75340	1	1.31e-01
protein N-linked glycosylation via asparagine	GO:0018279	P30101	1	1.36e-01
protein stabilization	GO:0050821	Q99497	1	1.41e-01
respiratory electron transport chain	GO:0022904	P06576	1	1.44e-01
negative regulation of cell migration	GO:0030336	P18206	1	1.48e-01
muscle contraction	GO:0006936	P18206	1	1.50e-01
positive regulation of neuron projection development	GO:0010976	P15531	1	1.63e-01
apoptotic signaling pathway	GO:0097190	O75340	1	1.67e-01
blood coagulation	GO:0007596	P11021,P18206	2	1.70e-01
central nervous system development	GO:0007417	P09211	1	1.75e-01
male gonad development	GO:0008584	Q13162	1	1.76e-01
cytoskeleton organization	GO:0007010	Q16555	1	1.79e-01
cell-matrix adhesion	GO:0007160	P18206	1	1.84e-01
response to estradiol	GO:0032355	P04083	1	1.84e-01

autophagy	GO:0006914	Q99497	1	1.86e-01
cellular response to lipopolysaccharide	GO:0071222	P09211	1	1.91e-01
protein dephosphorylation	GO:0006470	P30153	1	1.95e-01
G2/M transition of mitotic cell cycle	GO:0000086	P30153	1	2.02e-01
response to drug	GO:0042493	P04083,Q16555	2	2.13e-01
oxidation-reduction process	GO:0055114	Q13162,P32119,Q99497,P30041	4	2.14e-01
regulation of cell proliferation	GO:0042127	P04083	1	2.26e-01
cell adhesion	GO:0007155	P18206,P08865	2	2.36e-01
regulation of cell shape	GO:0008360	P15311	1	2.39e-01
regulation of cell cycle	GO:0051726	P11142	1	2.44e-01
positive regulation of gene expression	GO:0010628	P15311	1	2.51e-01
cell surface receptor signaling pathway	GO:0007166	P04083	1	2.56e-01
positive regulation of cell migration	GO:0030335	P11021	1	2.58e-01
post-translational protein modification	GO:0043687	P30101	1	2.65e-01
fibroblast growth factor receptor signaling pathway	GO:0008543	P30153	1	2.66e-01
lipid metabolic process	GO:0006629	P06576	1	2.85e-01
mRNA processing	GO:0006397	P11142	1	2.87e-01
DNA replication	GO:0006260	P35232	1	2.94e-01
negative regulation of endopeptidase activity	GO:0010951	P30740	1	2.97e-01
peptidyl-tyrosine dephosphorylation	GO:0035335	A6NDG6	1	3.04e-01
dephosphorylation	GO:0016311	A6NDG6	1	3.08e-01
nervous system development	GO:0007399	Q16555	1	3.71e-01
phosphorylation	GO:0016310	P55263	1	3.92e-01
negative regulation of transcription from RNA polymerase II promoter	GO:0000122	P35232,P06733	2	4.13e-01
spermatogenesis	GO:0007283	Q13162	1	5.09e-01
positive regulation of transcription from RNA polymerase II promoter	GO:0045944	P61978,Q99497	2	5.41e-01
intracellular protein transport	GO:0006886	O75340	1	5.67e-01
transcription from RNA polymerase II promoter	GO:0006366	P35232	1	5.92e-01
positive regulation of transcription, DNA-templated	GO:0045893	P35232	1	6.06e-01
transport	GO:0006810	P29373	1	6.13e-01
intracellular signal transduction	GO:0035556	P04792	1	6.36e-01
small GTPase mediated signal transduction	GO:0007264	P27348	1	6.42e-01
transcription, DNA-templated	GO:0006351	P61978,P06733,P11142	3	6.75e-01

G-protein coupled receptor signaling pathway	GO:0007186	O14908	1	8.02e-01
regulation of transcription, DNA-templated	GO:0006355	P35232,P29373,P30153	3	8.59e-01

Table S1B Cell Component (CC) of GO enrichment analysis

GO Name	GO ID	Genes	Count	P-value
extracellular vesicular exosome	GO:0070062	P08107,Q13162,P23381,P30740,P35527,P13645,P30101,P40227,P15531,P35232,P15311,P04792,O75340,P29373,P08670,P49368,P61978,P32119,P17987,P09960,Q15084,P05388,P38646,P40121,P04083,P27348,P13489,P04264,P09104,P11021,P28070,O14908,P09211,P41250,P18206,Q04695,Q99497,P08865,P30041,P06576,P06733,P05091,Q06323,P05787,P11142,Q16555,P15374	47	1.11e-44
cytosol	GO:0005829	P08107,P23381,P40227,P55263,P15531,P15311,P04792,P35998,P08670,P49368,P32119,P17987,P09960,P05388,P27348,P30153,P09104,P28070,O14908,P09211,P41250,P18206,Q99497,P08865,P30041,P06733,Q06323,P11142,P43686,Q16555	30	2.27e-17
cytoplasm	GO:0005737	P08107,P23381,P30740,P13645,P40227,P15531,P35232,P04792,O75340,P29373,P35998,P08670,P49368,P61978,P32119,P09960,P05388,P38646,P40121,P04083,P27348,P13489,O14908,P09211,P41250,Q04695,Q99497,P08865,P30041,P06733,Q06323,P05787,P43686,P15374	34	2.29e-11
membrane	GO:0016020	P30740,P35527,P13645,P15531,P35232,P15311,P35998,P55795,P61978,P31943,P05388,P27348,P30153,P04264,P11021,O14908,P08865,P30041,P06576,P06733,P11142,P43686,Q16555	23	7.92e-11
nucleus	GO:0005634	P08107,Q13162,P23381,P35527,P13645,P30101,P55263,P15531,P35232,P04792,O75340,P29373,P35998,P55795,P61978,P31943,P09960,P05388,P40121,P04083,P30153,P04264,P50213,P11021,P28070,P09211,P41250,Q99497,P08865,P06576,P06733,P05787,P11142,P43686,P15374	35	1.77e-08
proteasome complex	GO:0000502	P04792,P35998,P28070,Q06323,P43686	5	4.80e-08
extracellular space	GO:0005615	Q13162,P30740,P35527,P13645,P15311,P04792,P04083,P04264,P09104,P09211,P30041,P06733,P11142	13	4.15e-07
melanosome	GO:0042470	P30101,Q15084,P40121,P11021,P11142	5	7.57e-07
chaperonin-containing T-complex	GO:0005832	P40227,P49368,P17987	3	5.57e-06
intermediate filament	GO:0005882	P35527,P13645,P08670,Q04695,P05787	5	6.13e-06
zona pellucida receptor complex	GO:0002199	P40227,P49368,P17987	3	6.44e-06
cell body	GO:0044297	P40227,P15311,P49368,P17987	4	1.54e-05
nucleoplasm	GO:0005654	P35232,P35998,P55795,P61978,P31943,P28070,Q06323,P05787,P43686	9	8.54e-05
ribonucleoprotein complex	GO:0030529	P08107,P55795,P05388,P11142	4	1.19e-04
inclusion body	GO:0016234	P08107,P43686	2	5.00e-04
phosphopyruvate hydratase complex	GO:0000015	P09104,P06733	2	5.00e-04
mitochondrion	GO:0005739	P08107,Q13162,P15531,P35232,P38646,P30153,P50213,P09211,Q99497,P06576,Q16555	11	5.11e-04
proteasome accessory complex	GO:0022624	P35998,P43686	2	9.17e-04

keratin filament	GO:0045095	P13645,P04264,P05787	3	9.97e-04
mitochondrial matrix	GO:0005759	P50213,P41250,P06576,P05091	4	1.09e-03
TRAF2-GSTP1 complex	GO:0097057	P09211	1	1.65e-03
Schwann cell microvillus	GO:0097454	P15311	1	1.65e-03
mitochondrial proton-transporting ATP synthase, catalytic core	GO:0005754	P06576	1	1.65e-03
cytoskeleton	GO:0005856	P04792,P08670,P49368,P04264,P18206	5	1.80e-03
costamere	GO:0043034	P18206,P05787	2	1.91e-03
mitochondrial nucleoid	GO:0042645	P38646,P06576	2	2.11e-03
blood microparticle	GO:0072562	P08107,P04264,P11142	3	2.47e-03
angiogenin-PRI complex	GO:0032311	P13489	1	3.30e-03
microspike	GO:0044393	P15311	1	3.30e-03
endoplasmic reticulum lumen	GO:0005788	P30101,Q15084,P11021	3	3.38e-03
COP9 signalosome	GO:0008180	P08107,P11021	2	3.90e-03
endoplasmic reticulum-Golgi intermediate compartment	GO:0005793	Q15084,P11021	2	4.32e-03
endoplasmic reticulum chaperone complex	GO:0034663	P11021	1	4.95e-03
Prp19 complex	GO:0000974	P11142	1	6.59e-03
endoplasmic reticulum	GO:0005783	P08107,P30101,O75340,P29373,Q15084,P11021	6	6.61e-03
cell projection	GO:0042995	P08670,P61978	2	6.71e-03
microtubule	GO:0005874	P40227,P49368,P17987,Q16555	4	7.33e-03
catalytic step 2 spliceosome	GO:0071013	P61978,P31943	2	7.80e-03
cell-substrate junction	GO:0030055	P18206	1	8.24e-03
90S preribosome	GO:0030686	P08865	1	8.24e-03
plasma membrane	GO:0005886	P15311,P04792,P08670,P49368,Q15084,P04083,P04264,P09104,P09211,P18206,Q99497,P08865,P06576,P06733,P11142	15	8.42e-03
mitochondrial membrane	GO:0031966	P04083,P06576	2	1.00e-02
ruffle membrane	GO:0032587	P15531,P15311	2	1.04e-02
cell tip	GO:0051286	P15311	1	1.15e-02
cytosolic proteasome complex	GO:0031597	P43686	1	1.15e-02
astrocyte projection	GO:0097449	P15311	1	1.31e-02
clathrin-sculpted gamma-aminobutyric acid transport vesicle membrane	GO:0061202	P11142	1	1.31e-02
cell surface	GO:0009986	P30101,P04083,P11021,P06576	4	1.40e-02
acrosomal vesicle	GO:0001669	P40227,P17987	2	1.72e-02
cytoplasmic membrane-bounded vesicle	GO:0016023	O14908,P30041	2	1.94e-02
uropod	GO:0001931	P15311	1	2.29e-02
sarcolemma	GO:0042383	P04083,P05787	2	2.41e-02
F-actin capping protein complex	GO:0008290	P40121	1	2.45e-02
proteasome activator complex	GO:0008537	Q06323	1	2.45e-02

Z disc	GO:0030018	P04792,P05787	2	2.86e-02
nucleolus	GO:0005730	P15311,P29373,P61978,P09960,P38646,P40121,P40121,P41250,P11142,P43686	10	3.13e-02
mitochondrial proton-transporting ATP synthase complex	GO:0005753	P06576	1	3.41e-02
cytoplasmic ribonucleoprotein granule	GO:0036464	P05388	1	3.57e-02
microvillus membrane	GO:0031528	P15311	1	3.73e-02
protein complex	GO:0043234	P04083,P27348,P18206	3	3.76e-02
cornified envelope	GO:0001533	P04083	1	3.89e-02
pericentriolar material	GO:0000242	P17987	1	4.53e-02
M band	GO:0031430	P06733	1	4.53e-02
podosome	GO:0002102	P61978	1	4.84e-02
fascia adherens	GO:0005916	P18206	1	4.84e-02
basolateral plasma membrane	GO:0016323	P15311,P04083	2	4.97e-02
aggresome	GO:0016235	P08107	1	5.47e-02
cortical cytoskeleton	GO:0030863	P15311	1	5.47e-02
photoreceptor inner segment	GO:0001917	P09104	1	5.63e-02
vesicle membrane	GO:0012506	O14908	1	5.78e-02
adherens junction	GO:0005912	P18206	1	5.78e-02
nuclear heterochromatin	GO:0005720	P17987	1	6.09e-02
cell periphery	GO:0071944	Q04695	1	6.09e-02
dystrophin-associated glycoprotein complex	GO:0016010	P05787	1	6.41e-02
brush border	GO:0005903	O14908	1	6.41e-02
focal adhesion	GO:0005925	P15311,P18206	2	6.48e-02
proteasome core complex	GO:0005839	P28070	1	6.87e-02
cytosolic small ribosomal subunit	GO:0022627	P08865	1	6.87e-02
protein phosphatase type 2A complex	GO:0000159	P30153	1	7.02e-02
nuclear membrane	GO:0031965	O75340,P40121	2	7.22e-02
T-tubule	GO:0030315	P15311	1	7.79e-02
membrane raft	GO:0045121	P15311,Q99497	2	8.07e-02
microvillus	GO:0005902	P15311	1	8.25e-02
dendritic shaft	GO:0043198	O14908	1	8.40e-02
cell-cell adherens junction	GO:0005913	P18206	1	8.70e-02
actin filament	GO:0005884	P15311	1	8.70e-02
cytosolic large ribosomal subunit	GO:0022625	P05388	1	8.85e-02
cell leading edge	GO:0031252	P08670	1	1.04e-01
endocytic vesicle	GO:0030139	O14908	1	1.04e-01
chromosome, centromeric region	GO:0000775	P30153	1	1.06e-01
extrinsic component of	GO:0019898	P15311	1	1.06e-01

membrane				
actin cytoskeleton	GO:0015629	P15311,P18206	2	1.09e-01
perikaryon	GO:0043204	P09104	1	1.15e-01
cytoplasmic mRNA processing body	GO:0000932	P35998	1	1.23e-01
filopodium	GO:0030175	P15311	1	1.40e-01
integral component of endoplasmic reticulum membrane	GO:0030176	P11021	1	1.44e-01
endoplasmic reticulum membrane	GO:0005789	O75340,Q15084,P11021	3	1.52e-01
secretory granule	GO:0030141	P41250	1	1.54e-01
centriole	GO:0005814	P08107	1	1.55e-01
intermediate filament cytoskeleton	GO:0045111	P08670	1	1.60e-01
PML body	GO:0016605	Q99497	1	1.73e-01
spliceosomal complex	GO:0005681	P11142	1	1.75e-01
cytoplasmic vesicle membrane	GO:0030659	P27348	1	1.82e-01
cell-cell junction	GO:0005911	P18206	1	1.84e-01
dendritic spine	GO:0043197	O14908	1	1.87e-01
nuclear matrix	GO:0016363	P05787	1	1.92e-01
midbody	GO:0030496	P11021	1	1.95e-01
centrosome	GO:0005813	P15531,P17987	2	1.96e-01
ruffle	GO:0001726	P15311	1	2.08e-01
peroxisome	GO:0005777	P08670	1	2.16e-01
spindle	GO:0005819	P04792	1	2.16e-01
apical part of cell	GO:0045177	P15311	1	2.18e-01
cytoplasmic vesicle	GO:0031410	O75340	1	2.24e-01
synaptic vesicle	GO:0008021	O14908	1	2.24e-01
cilium	GO:0005929	P04083	1	2.29e-01
growth cone	GO:0030426	Q16555	1	2.36e-01
nuclear chromatin	GO:0000790	P61978	1	2.49e-01
cell cortex	GO:0005938	O14908	1	2.59e-01
nuclear speck	GO:0016607	P08107	1	2.70e-01
microtubule cytoskeleton	GO:0015630	P30153	1	2.72e-01
endosome	GO:0005768	O75340	1	2.80e-01
perinuclear region of cytoplasm	GO:0048471	P08107,P15531	2	3.29e-01
lysosome	GO:0005764	P30041	1	3.53e-01
axon	GO:0030424	Q16555	1	3.78e-01
apical plasma membrane	GO:0016324	P15311	1	4.65e-01
dendrite	GO:0030425	Q16555	1	4.83e-01
neuronal cell body	GO:0043025	Q16555	1	5.23e-01
mitochondrial inner membrane	GO:0005743	P35232	1	5.48e-01
cell junction	GO:0030054	P61978	1	5.68e-01

intracellular	GO:0005622	P09211,P11142	2	7.37e-01
extracellular region	GO:0005576	P04083,P18206	2	8.19e-01
Golgi apparatus	GO:0005794	P17987	1	8.60e-01
integral component of plasma membrane	GO:0005887	P35232	1	8.64e-01

Table S1C Molecular Functions (MF) of GO enrichment analysis

GO Name	GO ID	Genes	Count	P-value
protein binding	GO:0005515	P08107,Q13162,P23381,P30101,P15531,P35232,P15311,P04792,O75340,P35998,P08670,P49368,P61978,P31943,P17987,O95816,Q15084,P05388,P38646,P04083,P27348,P13489,P30153,P04264,P11021,O14908,P09211,P18206,Q04695,Q99497,P08865,P30041,P06576,P06733,P05787,P11142,P43686,Q16555,P15374	39	2.89e-18
poly(A) RNA binding	GO:0044822	P08107,P30101,P40227,P55263,P15531,P15311,P04792,P55795,P49368,P61978,P31943,P17987,P09960,P05388,P38646,P08865,P06733,P11142	18	2.54e-14
unfolded protein binding	GO:0051082	P08107,P40227,P49368,P17987,P38646,P11021,P11142	7	3.46e-08
ATPase activity	GO:0016887	P08107,P35998,P11021,P06576,P11142,P43686	6	7.52e-07
enzyme binding	GO:0019899	P08107,P35232,P38646,P11021,Q99497,P11142	6	1.20e-05
thioredoxin peroxidase activity	GO:0008379	Q13162,P32119	2	1.39e-05
peroxiredoxin activity	GO:0051920	Q99497,P30041	2	1.39e-05
scaffold protein binding	GO:0097110	P08670,Q99497,P05787	3	3.25e-05
magnesium ion binding	GO:0000287	P15531,P50213,P09104,A6NDG6,P06733	5	3.06e-04
phosphopyruvate hydratase activity	GO:0004634	P09104,P06733	2	4.32e-04
antioxidant activity	GO:0016209	P32119,P30041	2	4.77e-04
single-stranded DNA binding	GO:0003697	P15531,P61978,Q99497	3	4.98e-04
peptidase activity	GO:0008233	P09960,Q99497,P15374	3	6.41e-04
ATPase activity, coupled	GO:0042623	P08107,P11142	2	8.53e-04
ATP binding	GO:0005524	P08107,P23381,P40227,P55263,P15531,P35998,P49368,P17987,P38646,P11021,P41250,P06576,P11142,P43686	14	8.56e-04
protein disulfide isomerase activity	GO:0003756	P30101,Q15084	2	1.05e-03
small protein activating enzyme binding	GO:0044388	Q99497	1	1.53e-03
adenosine kinase activity	GO:0004001	P55263	1	1.53e-03
S-nitrosoglutathione binding	GO:0035730	P09211	1	1.53e-03
dinitrosyl-iron complex binding	GO:0035731	P09211	1	1.53e-03
cuprous ion binding	GO:1903136	Q99497	1	1.53e-03
leukotriene-A4 hydrolase activity	GO:0004463	P09960	1	1.53e-03
small protein conjugating enzyme binding	GO:0044390	Q99497	1	1.53e-03
glycine-tRNA ligase activity	GO:0004820	P41250	1	1.53e-03
cupric ion binding	GO:1903135	Q99497	1	1.53e-03
nitric oxide binding	GO:0070026	P09211	1	1.53e-03
structural constituent of	GO:0005200	P35527,P08670,Q04695	3	1.74e-03

cytoskeleton				
heat shock protein binding	GO:0031072	P08107,P11142	2	2.81e-03
structural molecule activity	GO:0005198	P04083,P04264,P18206,P05787	4	2.96e-03
ubiquitin binding	GO:0043130	P04792,P15374	2	3.03e-03
dihydropyrimidinase activity	GO:0004157	Q16555	1	3.07e-03
oxidoreductase activity, acting on peroxide as acceptor	GO:0016684	Q99497	1	3.07e-03
ribonuclease inhibitor activity	GO:0008428	P13489	1	3.07e-03
kinase regulator activity	GO:0019207	P09211	1	3.07e-03
superoxide dismutase copper chaperone activity	GO:0016532	Q99497	1	3.07e-03
phosphoglycolate phosphatase activity	GO:0008967	A6NDG6	1	3.07e-03
ribosome binding	GO:0043022	P11021,P08865	2	3.15e-03
laminin receptor activity	GO:0005055	P08865	1	4.60e-03
binding, bridging	GO:0060090	O75340	1	4.60e-03
chaperone binding	GO:0051087	O95816,P11021	2	5.82e-03
epoxide hydrolase activity	GO:0004301	P09960	1	6.13e-03
protein kinase C inhibitor activity	GO:0008426	P04792	1	6.13e-03
ubiquitin-specific protease binding	GO:1990381	Q99497	1	6.13e-03
double-stranded RNA binding	GO:0003725	P08107,P08670	2	6.77e-03
calcium-dependent protein binding	GO:0048306	O75340,P04083	2	7.61e-03
phospholipase A2 inhibitor activity	GO:0019834	P04083	1	7.65e-03
endopeptidase activator activity	GO:0061133	Q06323	1	7.65e-03
nucleotide phosphatase activity, acting on free nucleotides	GO:0098519	A6NDG6	1	7.65e-03
glycoprotein binding	GO:0001948	P08670,P11021	2	7.96e-03
isocitrate dehydrogenase (NAD+) activity	GO:0004449	P50213	1	9.18e-03
JUN kinase binding	GO:0008432	P09211	1	1.07e-02
structural constituent of epidermis	GO:0030280	P13645	1	1.07e-02
retinoid binding	GO:0005501	P29373	1	1.22e-02
aldehyde dehydrogenase [NAD(P)+] activity	GO:0004030	P05091	1	1.22e-02
intermediate filament binding	GO:0019215	P15531	1	1.22e-02
misfolded protein binding	GO:0051787	P11021	1	1.22e-02
identical protein binding	GO:0042802	P15531,P04792,P08670,Q99497	4	1.30e-02

MHC class II protein binding	GO:0042289	Q04695	1	1.37e-02
aldehyde dehydrogenase (NAD) activity	GO:0004029	P05091	1	1.37e-02
protein N-terminus binding	GO:0047485	P08107,P27348	2	1.38e-02
ribosomal small subunit binding	GO:0043024	P15531	1	1.52e-02
protein domain specific binding	GO:0019904	P15311,P27348,P11021	3	1.59e-02
actin binding	GO:0003779	P40121,O14908,P18206,P18206	4	1.63e-02
protein binding involved in protein folding	GO:0044183	P08107	1	1.83e-02
retinal binding	GO:0016918	P29373	1	1.83e-02
poly(U) RNA binding	GO:0008266	P31943	1	1.83e-02
MHC class II receptor activity	GO:0032395	Q04695	1	2.13e-02
dystroglycan binding	GO:0002162	P18206	1	2.13e-02
receptor binding	GO:0005102	P04083,O14908,Q99497	3	2.17e-02
MHC class I protein binding	GO:0042288	P06576	1	2.28e-02
MHC class II protein complex binding	GO:0023026	P11142	1	2.58e-02
tryptophan-tRNA ligase activity	GO:0004830	P23381	1	2.58e-02
phospholipase C activity	GO:0004629	P30101	1	2.73e-02
retinol binding	GO:0019841	P29373	1	2.73e-02
deoxyribonuclease activity	GO:0004536	P15531	1	2.73e-02
lipopolysaccharide binding	GO:0001530	P28070	1	3.18e-02
protein homodimerization activity	GO:0042803	O75340,P04083,O14908,Q99497	4	3.45e-02
alpha-catenin binding	GO:0045294	P18206	1	3.62e-02
retinoic acid binding	GO:0001972	P29373	1	3.77e-02
cytokine binding	GO:0019955	Q99497	1	3.77e-02
ubiquitin protein ligase binding	GO:0031625	P08107,P11021	2	3.80e-02
proton-transporting ATP synthase activity, rotational mechanism	GO:0046933	P06576	1	3.92e-02
phospholipase A2 activity	GO:0004623	P30041	1	3.92e-02
protein kinase binding	GO:0019901	P15531,P04792,Q16555	3	3.93e-02
RNA binding	GO:0003723	P55795,P61978,P31943,P05388	4	4.07e-02
proton-transporting ATPase activity, rotational mechanism	GO:0046961	P06576	1	4.51e-02
structural constituent of eye lens	GO:0005212	P08670	1	4.51e-02
myosin binding	GO:0017022	O14908	1	4.65e-02
virus receptor activity	GO:0001618	P08107	1	5.24e-02

glutathione peroxidase activity	GO:0004602	P30041	1	5.38e-02
ubiquitin thiolesterase activity	GO:0004221	P15374	1	5.67e-02
laminin binding	GO:0043236	P08865	1	5.67e-02
protein phosphatase type 2A regulator activity	GO:0008601	P30153	1	5.82e-02
androgen receptor binding	GO:0050681	Q99497	1	5.82e-02
peroxidase activity	GO:0004601	Q99497	1	5.82e-02
nucleoside diphosphate kinase activity	GO:0004550	P15531	1	6.68e-02
gamma-tubulin binding	GO:0043015	P15531	1	6.83e-02
glutathione transferase activity	GO:0004364	P09211	1	6.83e-02
cell adhesion molecule binding	GO:0050839	P15311	1	6.97e-02
protein dimerization activity	GO:0046983	O75340,P41250	2	6.98e-02
aminopeptidase activity	GO:0004177	P09960	1	7.68e-02
protein kinase C binding	GO:0005080	P04792	1	8.25e-02
threonine-type endopeptidase activity	GO:0004298	P28070	1	8.25e-02
cadherin binding	GO:0045296	P18206	1	8.53e-02
calcium-dependent cysteine-type endopeptidase activity	GO:0004198	O75340	1	8.53e-02
protein serine/threonine phosphatase activity	GO:0004722	P30153	1	9.51e-02
transmembrane transporter activity	GO:0022857	P06576	1	9.51e-02
transporter activity	GO:0005215	P29373,P06576	2	9.56e-02
RNA polymerase II regulatory region sequence-specific DNA binding	GO:0000977	P15531	1	9.65e-02
protein binding, bridging	GO:0030674	P04083	1	9.79e-02
phosphotransferase activity, alcohol group as acceptor	GO:0016773	P55263	1	9.93e-02
NAD binding	GO:0051287	P50213	1	1.25e-01
histone deacetylase binding	GO:0042826	P35232	1	1.25e-01
beta-catenin binding	GO:0008013	P18206	1	1.31e-01
electron carrier activity	GO:0009055	P05091	1	1.31e-01
calcium-dependent phospholipid binding	GO:0005544	P04083	1	1.43e-01
phospholipid binding	GO:0005543	P04083	1	1.44e-01
sequence-specific DNA binding RNA polymerase II	GO:0000981	P35232	1	1.50e-01

transcription factor activity				
cysteine-type endopeptidase activity	GO:0004197	P30101	1	1.52e-01
mRNA binding	GO:0003729	Q99497	1	1.53e-01
structural constituent of ribosome	GO:0003735	P05388,P08865	2	1.54e-01
ubiquitin-specific protease activity	GO:0004843	P15374	1	1.65e-01
PDZ domain binding	GO:0030165	O14908	1	1.83e-01
metallopeptidase activity	GO:0008237	P09960	1	1.85e-01
double-stranded DNA binding	GO:0003690	Q99497	1	2.02e-01
RNA polymerase II core promoter proximal region sequence-specific DNA binding	GO:0000978	P61978	1	2.04e-01
serine-type endopeptidase inhibitor activity	GO:0004867	P30740	1	2.20e-01
antigen binding	GO:0003823	P30153	1	2.22e-01
RNA polymerase II core promoter proximal region sequence-specific DNA binding transcription factor activity involved in positive regulation of transcription	GO:0001077	P61978	1	2.33e-01
actin filament binding	GO:0051015	P15311	1	2.46e-01
protein C-terminus binding	GO:0008022	P08670	1	2.86e-01
protein tyrosine phosphatase activity	GO:0004725	A6NDG6	1	2.94e-01
transcription regulatory region DNA binding	GO:0044212	P35232	1	3.05e-01
transcription corepressor activity	GO:0003714	P06733	1	3.64e-01
carbohydrate binding	GO:0030246	P04264	1	3.75e-01
protein complex binding	GO:0032403	P05787	1	3.88e-01
receptor activity	GO:0004872	P04264	1	3.88e-01
nucleotide binding	GO:0000166	P55795,P31943	2	3.93e-01
calcium ion binding	GO:0005509	O75340,P04083,P11021	3	4.04e-01
transcription factor binding	GO:0008134	Q99497	1	4.13e-01
protein heterodimerization activity	GO:0046982	P30153	1	6.05e-01
GTP binding	GO:0005525	P15531	1	7.88e-01
sequence-specific DNA binding transcription factor activity	GO:0003700	P06733	1	9.48e-01

DNA binding	GO:0003677	P06733	1	9.81e-01
zinc ion binding	GO:0008270	P09960	1	9.83e-01
metal ion binding	GO:0046872	P55263	1	9.88e-01

Table S2 The KEGG pathway analysis of differentially expressed proteins

Pathway name	Pathway id	Genes uid	Count	Pvalue
Antigen processing and presentation	hsa04612	P08107,P30101,P11021,Q06323,P11142	5	7.53e-05
Proteasome	hsa03050	P35998,P28070,Q06323,P43686	4	8.88e-05
Epstein-Barr virus infection	hsa05169	P08107,P04792,P35998,P08670,P27348,P11142,P43686	7	9.31e-05
Protein processing in endoplasmic reticulum	hsa04141	P08107,P30101,O95816,Q15084,P11021,P11142	6	2.67e-04
Glycolysis / Gluconeogenesis	hsa00010	P09104,P06733,P05091	3	5.48e-03
RNA degradation	hsa03018	P38646,P09104,P06733	3	7.25e-03
Amoebiasis	hsa05146	P30740,P04792,P18206	3	2.14e-02
Spliceosome	hsa03040	P08107,P61978,P11142	3	3.44e-02
Legionellosis	hsa05134	P08107,P11142	2	3.62e-02
Pathogenic Escherichia coli infection	hsa05130	P15311,P27348	2	3.62e-02
Aminoacyl-tRNA biosynthesis	hsa00970	P23381,P41250	2	5.04e-02
Phenylalanine metabolism	hsa00360	P30041	1	9.43e-02
Estrogen signaling pathway	hsa04915	P08107,P11142	2	1.04e-01
HIF-1 signaling pathway	hsa04066	P09104,P06733	2	1.14e-01
Protein export	hsa03060	P11021	1	1.19e-01
Oocyte meiosis	hsa04114	P27348,P30153	2	1.21e-01
Glyoxylate and dicarboxylate metabolism	hsa00630	A6NDG6	1	1.29e-01
Leukocyte transendothelial migration	hsa04670	P15311,P18206	2	1.36e-01
Ascorbate and aldarate metabolism	hsa00053	P05091	1	1.38e-01
Histidine metabolism	hsa00340	P05091	1	1.43e-01
Toxoplasmosis	hsa05145	P08107,P11142	2	1.44e-01
beta-Alanine metabolism	hsa00410	P05091	1	1.52e-01
Citrate cycle (TCA cycle)	hsa00020	P50213	1	1.52e-01
Propanoate metabolism	hsa00640	P05091	1	1.62e-01
MAPK signaling pathway	hsa04010	P08107,P04792,P11142	3	1.65e-01
Measles	hsa05162	P08107,P11142	2	1.67e-01
Ribosome	hsa03010	P05388,P08865	2	1.69e-01
Pentose and glucuronate interconversions	hsa00040	P05091	1	1.71e-01
Prion diseases	hsa05020	P11021	1	1.80e-01
Parkinson's disease	hsa05012	Q99497,P06576	2	1.84e-01
Tryptophan metabolism	hsa00380	P05091	1	1.98e-01
Pyruvate metabolism	hsa00620	P05091	1	2.02e-01
Hippo signaling pathway	hsa04390	P27348,P30153	2	2.06e-01
Fatty acid degradation	hsa00071	P05091	1	2.15e-01

Valine, leucine and isoleucine degradation	hsa00280	P05091	1	2.15e-01
MicroRNAs in cancer	hsa05206	P15311,P08670,P61978	3	2.20e-01
Purine metabolism	hsa00230	P55263,P15531	2	2.41e-01
Glutathione metabolism	hsa00480	P09211	1	2.45e-01
Lysine degradation	hsa00310	P05091	1	2.45e-01
Influenza A	hsa05164	P08107,P11142	2	2.57e-01
Glycerolipid metabolism	hsa00561	P05091	1	2.62e-01
Staphylococcus aureus infection	hsa05150	P13645	1	2.78e-01
Arginine and proline metabolism	hsa00330	P05091	1	2.78e-01
Long-term depression	hsa04730	P30153	1	2.82e-01
VEGF signaling pathway	hsa04370	P04792	1	2.86e-01
Shigellosis	hsa05131	P18206	1	2.86e-01
Arachidonic acid metabolism	hsa00590	P09960	1	2.98e-01
Endocytosis	hsa04144	P08107,P11142	2	3.06e-01
Viral carcinogenesis	hsa05203	P61978,P27348	2	3.13e-01
Drug metabolism - cytochrome P450	hsa00982	P09211	1	3.13e-01
Thyroid hormone synthesis	hsa04918	P11021	1	3.28e-01
Regulation of actin cytoskeleton	hsa04810	P15311,P18206	2	3.31e-01
Adherens junction	hsa04520	P18206	1	3.32e-01
Metabolism of xenobiotics by cytochrome P450	hsa00980	P09211	1	3.36e-01
Gastric acid secretion	hsa04971	P15311	1	3.39e-01
Bacterial invasion of epithelial cells	hsa05100	P18206	1	3.43e-01
TGF-beta signaling pathway	hsa04350	P30153	1	3.58e-01
Chemical carcinogenesis	hsa05204	P09211	1	3.58e-01
Prostate cancer	hsa05215	P09211	1	3.89e-01
mRNA surveillance pathway	hsa03015	P30153	1	3.96e-01
Chagas disease (American trypanosomiasis)	hsa05142	P30153	1	4.38e-01
Pyrimidine metabolism	hsa00240	P15531	1	4.41e-01
Cell cycle	hsa04110	P27348	1	4.97e-01
Axon guidance	hsa04360	Q16555	1	5.06e-01
Dopaminergic synapse	hsa04728	P30153	1	5.17e-01
Oxidative phosphorylation	hsa00190	P06576	1	5.22e-01
Hepatitis C	hsa05160	P30153	1	5.22e-01
Tight junction	hsa04530	P30153	1	5.25e-01
Hepatitis B	hsa05161	P27348	1	5.56e-01
Adrenergic signaling in cardiomyocytes	hsa04261	P30153	1	5.63e-01
PI3K-Akt signaling pathway	hsa04151	P27348,P30153	2	5.73e-01
Alzheimer's disease	hsa05010	P06576	1	6.08e-01

Tuberculosis	hsa05152	P38646	1	6.37e-01
Huntington's disease	hsa05016	P06576	1	6.39e-01
Herpes simplex infection	hsa05168	P61978	1	6.49e-01
Focal adhesion	hsa04510	P18206	1	6.83e-01
Proteoglycans in cancer	hsa05205	P15311	1	7.16e-01
Pathways in cancer	hsa05200	P09211	1	8.42e-01
